# A novel approach to UAV control: Fuzzy PID-based ANFIS (FPIDANFIS)

**DOI:** 10.1371/journal.pone.0355158

**Published:** 2026-08-07

**Authors:** Nigatu Wanore Madebo, Feleke Tsegaye Yareshe, Lebsework Negash Lemma

**Affiliations:** 1 Information Network Security Administration (INSA), Addis Ababa, Ethiopia; 2 School of Electrical and Computer Engineering, Addis Ababa University, Addis Ababa, Ethiopia; Industrial University of Ho Chi Minh City, VIET NAM

## Abstract

This paper presents a novel hybrid control strategy, the Fuzzy PID based Adaptive Neuro-Fuzzy Inference System (FPIDANFIS), designed to enhance the trajectory tracking performance of Unmanned Aerial Vehicles (UAVs). The distinct contribution of this work lies in its data-driven design approach: historical input–output data from a baseline Fuzzy PID (FPID) controller using tracking error and its derivative as inputs and FPID control signals as outputs are leveraged to train an ANFIS model that autonomously generates the adaptive FPIDANFIS controller. This methodology differs from existing studies by enabling the new controller to inherit the baseline controller’s behavior while introducing adaptive capabilities not present in conventional FPID frameworks. A dataset of 5001 samples is generated using MATLAB’s Neuro-Fuzzy Designer toolbox for offline training with a hybrid learning algorithm and Gaussian membership functions. Once trained, FPIDANFIS dynamically tunes control actions in real time, improving robustness and adaptability under uncertainty. The controller is evaluated across three scenarios: nominal trajectory tracking, tracking with input disturbances, and tracking under parameter variations. Performance is quantified using the Integral of Time-weighted Absolute Error (ITAE). Simulation results show that FPIDANFIS outperforms the baseline FPID controller, achieving a 42% ITAE reduction under disturbances and a 22.12% reduction under parameter variations. The proposed method introduces a data-driven, adaptive extension of the FPID framework and demonstrates significant improvements in UAV trajectory tracking accuracy, making it suitable for applications requiring precise and robust maneuverability.

## 1 Introduction

The adoption of Unmanned Aerial Vehicles (UAVs) has rapidly expanded across numerous civil applications, including real-time monitoring, remote sensing, search and rescue, security, precision agriculture, and infrastructure inspection. The integration of smart UAV technology has significantly transformed these applications by reducing operational risks and costs. Among various domains, civil infrastructure is projected to dominate the UAV market, contributing to a global market value exceeding 45 billion dollars [1]. Ensuring safe, reliable, and efficient UAV operations necessitates advanced automation and control systems, which encompass navigation, flight control, mission planning, and emergency handling.

Despite the significant progress in UAV automation, several challenges persist, particularly in multi-UAV coordination, communication, and collision avoidance [2]. Effective flight control must ensure feasible maneuvers under adverse conditions, while navigation systems should leverage sensor fusion techniques to operate in dynamic environments, even during sensor failures. Fail-safe mechanisms, such as emergency landing protocols and return-to-home functionalities, are crucial for mitigating operational risks. Additionally, recent advancements in artificial intelligence (AI) and machine learning (ML) offer promising solutions for enhancing UAV autonomy and adaptive decision-making [3]. Quadrotors, in particular, present substantial control challenges due to their under-actuated dynamics, high sensitivity to external disturbances, and inherent instability. The complexity of motion planning in unknown and dynamic environments further amplifies the need for robust and adaptive control strategies [4,5]. Conventional Proportional-Integral-Derivative (PID) controllers, while widely used due to their simplicity, often struggle to maintain stability and performance in nonlinear and rapidly changing environments. As a result, intelligent control techniques have emerged as viable alternatives [6]. Fuzzy Logic Controllers (FLCs) have gained increasing prominence in UAV control owing to their effectiveness in managing system uncertainties and nonlinear dynamics [7–9]. Building on this foundation, the integration of fuzzy logic with neural network learning in Adaptive Neuro-Fuzzy Inference Systems (ANFIS) has enabled the design of intelligent adaptive controllers that combine heuristic reasoning with data-driven learning mechanisms [10–12]. ANFIS-based control architectures are particularly appealing for UAV applications, as they exhibit strong anti-interference capabilities, rapid convergence characteristics, and enhanced adaptability in complex and dynamic operating environments [13–15]. These strengths have motivated extensive research on ANFIS-enhanced UAV controllers, especially for trajectory tracking tasks subject to disturbances, modeling uncertainties, and parameter variations [16–22]. By leveraging both the structured reasoning of fuzzy systems and the adaptive learning capability of neural networks, ANFIS-based hybrid control methods offer a compelling pathway toward improving precision, robustness, and autonomy in aerial robotic platforms [23,24].

In summary, the existing literature underscores that hybrid control algorithms particularly those integrating fuzzy logic and learning-based adaptation play a vital role in achieving reliable and high-performance UAV trajectory tracking in the presence of disturbances and uncertainties.

### 1.1 Related work

Recent advancements in quadrotor UAV control have increasingly focused on overcoming the challenges posed by their nonlinear, underactuated, and inherently unstable dynamics. Although proportional–integral–derivative (PID) controllers remain widely adopted because of their structural simplicity, they frequently lack the robustness and adaptability required for complex real-world conditions characterized by parameter variations and external disturbances. To address these limitations, researchers have progressively shifted toward more advanced control algorithms, with particular emphasis on adaptive fuzzy-based methods [25–29]. Fuzzy PID-based adaptive controllers have demonstrated notable improvements in robustness, adaptability, and tracking accuracy in UAV trajectory control [30–36]. Building on these advances, hybrid strategies that integrate fuzzy logic with neural networks, particularly the Adaptive Neuro-Fuzzy Inference System (ANFIS), have emerged to further strengthen adaptability and learning capability [37]. The versatility of fuzzy PID controllers has also been validated in multi-joint robotic arm systems, where their ability to manage highly coupled dynamics while ensuring stability has been emphasized [38]. In [39], an ANFIS-based task-offloading model for mobile edge computing was proposed to address dynamic and resource-constrained environments. Implemented using MATLAB’s Neuro-Fuzzy Toolbox and evaluated through a 70% training and 30% testing split, the model achieved a low error rate of 0.29505, underscoring its effectiveness. Similarly, [40] introduced an ANFIS-based autonomous UAV flight controller employing three fuzzy logic modules to regulate altitude, speed, and roll angle. The system was modeled using MATLAB and the Aerosim Aeronautical Simulation Block Set and validated through Microsoft Flight Simulator and FlightGear, illustrating its suitability for rapid nonlinear UAV modeling. For fixed-wing UAVs, [41] presented an ANFIS-based heading control technique that combined fuzzy reasoning with neural learning procedures. Implemented on an onboard microcontroller equipped with real-time navigation sensors, the method enabled fully autonomous flight. Expanding on these developments, [42] compared a genetic algorithm–optimized self-tuning PID controller with an ANFIS-based controller using the Aerosonde UAV model in Simulink/MATLAB. Simulation results revealed significantly enhanced robustness from the ANFIS controller, particularly under windy conditions. Hybrid control architectures have also gained attention. In [43], a backstepping-based PID and ANFIS hybrid controller was designed for UAV formation control in cluttered environments. MATLAB Simulink simulations confirmed its capability to maintain leader–follower formations despite obstacles. A broader review in [44] further highlighted the extensive applicability of ANFIS across engineering domains, particularly within power electronics, where it contributes to power quality improvement in multilevel converters.

Beyond aerial robotics, ANFIS has been employed in automotive and energy systems. In [45], an ANFIS-based controller for anti-lock braking systems (ABS) was designed to optimize wheel slip and reduce braking distance, outperforming conventional PID and fuzzy-tuned PID controllers. Likewise, [46] developed an ANFIS-based bidirectional power management strategy for plug-in electric vehicles (PEVs), improving energy exchange efficiency between the grid and vehicle batteries while reducing harmonic distortion. Several additional studies have extended ANFIS applications to industrial control and UAV trajectory tracking. For instance, [47] implemented an ANFIS-based controller to improve accuracy in spray drying systems, resulting in increased thermal efficiency and product consistency. A trajectory tracking control strategy using an uncertainty and disturbance estimator (UDE) for quadrotors was presented in [48], while an adaptive trajectory tracking controller for quadrotor micro aerial vehicles (MAVs) was explored in [49]. To further mitigate external disturbances, adaptive disturbance compensation strategies were proposed in [50,51], followed by adaptive robust trajectory tracking methods in [52–54]. An ANFIS-based sliding mode control (ANFIS–SMC) scheme was introduced in [55] for UAV trajectory tracking and obstacle avoidance. Comparative simulations demonstrated that ANFIS–SMC significantly reduced tracking errors and improved operational safety—achieving a 0% collision probability compared to 33.33% for conventional SMC—albeit at the cost of increased computational time.

More recently, ANFIS has been employed directly for UAV trajectory tracking [56], where adaptive optimization of control parameters was achieved using an input–output dataset derived from a fuzzy PID (FPID) controller. This approach improves reference tracking accuracy while ensuring adaptability to nonlinear flight dynamics. Despite these advances, achieving consistently efficient reference tracking in quadrotor UAVs using ANFIS-based controllers requires precise and systematic tuning of control parameters across diverse operating conditions. Traditional PID and Fuzzy PID (FPID) controllers often struggle to address the strong nonlinearities inherent in quadrotor systems. Motivated by these gaps, this study proposes a trajectory-tracking control strategy based on a Fuzzy PID–based ANFIS (FPIDANFIS) controller. By leveraging the input–output characteristics of an FPID controller, the proposed method adaptively tunes control parameters to deliver robust and accurate trajectory tracking under nonlinear and dynamically changing flight conditions (Table 1).

**Table 1:** Summary of related work and identified research gaps.

Ref.	Method / Controller	Application Area	Key Contribution / Findings	Research Gap
[25–29]	Adaptive Fuzzy Controllers	Quadrotor UAV	Improved robustness and adaptability over classical PID.	Lacks learning-based tuning for real-time adaptation.
[30–36]	Fuzzy PID Controllers	UAV trajectory tracking	Enhanced control accuracy and disturbance rejection.	Limited self-tuning online in highly dynamic environments.
[37]	ANFIS	UAV control, nonlinear systems	Combines neural learning with fuzzy inference.	Often static; needs improved real-time adaptation.
[38]	Fuzzy PID Control	Multi-joint robotic arms	Effective for complex joint dynamics.	Not optimized for highly nonlinear aerial vehicles.
[39]	ANFIS Task Offloading	Mobile Edge Computing	Achieved low error rate (0.29505).	Not designed for UAV dynamic control or real-time tracking.
[40]	ANFIS Flight Controller	Autonomous UAV flight	Three-module fuzzy controller validated in simulation.	Tuning not optimized for strong disturbances or abrupt changes.
[41]	ANFIS Heading Control	Fixed-wing UAV	Real-time implementation with sensors and microcontroller.	Limited for complex 3D quadrotor trajectories.
[42]	GA-PID + ANFIS Controller	Aerosonde UAV	ANFIS outperformed GA-PID under wind.	No hybrid adaptive with disturbance observers.
[43]	Backstepping PID + ANFIS Hybrid	UAV formation control	Improved leader–follower tracking.	High computational cost; not for fast maneuvers.
[44]	Survey of ANFIS Applications	Engineering, power electronics	Highlights ANFIS advantages for nonlinear systems.	Lacks UAV trajectory tracking focus.
[45]	ANFIS-PID Controller	ABS	Reduced stopping distance, improved slip control.	Not tested in fast aerial systems.
[46]	ANFIS Power Management	PEVs	Improved grid–battery power exchange.	Not transferable to UAV control.
[47]	ANFIS Controller	Spray drying system	Improved thermal efficiency.	No application in fast aerial dynamics.
[48]	UDE-based Trajectory Control	Quadrotor UAVs	Efficient tracking under uncertainty.	Does not include adaptive fuzzy learning.
[49]	Adaptive Trajectory Tracking	Quadrotor MAVs	Improved robustness to uncertainty.	Lacks fuzzy-neural combination.
[50,51]	Adaptive Disturbance Compensation	UAV control	Effective disturbance rejection.	No integration with ANFIS/FPID.
[52–54]	Adaptive Robust Tracking	Quadrotor UAVs	Strong robustness to uncertainties.	No data-driven adaptive parameter optimization.
[55]	ANFIS-SMC Controller	UAV + obstacle avoidance	Reduced tracking error, 0% collision probability.	Higher computational load, slower execution.
[56]	ANFIS with FPID Dataset	Quadrotor UAV tracking	Adaptive control via FPID learning; improved accuracy.	Lacks unified FPID–ANFIS hybrid tuned in real-time.
**Proposed Work**		**Quadrotor UAV tracking**	**Combines FPID + ANFIS for dynamic parameter tuning.**	**Enables data-driven self-tuning of state parameters from baseline controller data for robust trajectory tracking.**

### 1.2 Contribution

The key contributions of this work include:

- Introduction of a novel control approach(FPIDANFIS) for quadrotor trajectory task.
- Development of an adaptive UAV controller utilizing learning-based generalization achieved through offline training that integrates a Fuzzy PID controller with an Adaptive Neuro-Fuzzy Inference System (ANFIS) to enhance the robustness of trajectory tracking.
- Evaluation of FPIDANFIS under various operating conditions, including nominal tracking, input disturbances, and parameter variations, using the Integral of Time-weighted Absolute Error (ITAE) criterion.
- Demonstrate enhanced adaptability and robustness using FPID as the baseline controller.

The proposed FPIDANFIS approach contributes to the development of intelligent UAV control frameworks, enabling enhanced maneuverability, stability, and resilience in complex environments.

### 1.3 Methodology

The development of the FPIDANFIS control system for UAV trajectory tracking follows a structured approach, comprising the following key stages:

- **Mathematical Modeling:** A nonlinear quadrotor mathematical model in an X-configuration is adopted to accurately represent UAV dynamics. The model incorporates six-degree-of-freedom (6-DOF) dynamics, including translational and rotational motions.
- **FPID Controller Design:** A Fuzzy PID (FPID) controller is implemented for six-state dynamic control of the UAV. This controller serves as a reference(baseline) for generating training data for the ANFIS-based control system.
- **Training Data Generation:** The training dataset for ANFIS is generated using input-output data obtained from the FPID controller. The input variables consist of the control error and its derivative, while the output corresponds to the FPID control signal.
- **FPIDANFIS Controller Development:** The proposed FPIDANFIS controller integrates the adaptive neuro-fuzzy inference system (ANFIS) with FPID control to enhance UAV trajectory tracking performance. Gaussian membership functions are employed due to their smooth interpolation and computational efficiency. The control system architecture, illustrated in Fig 8, utilizes error and the derivative of error as inputs, while the FPID-generated control actions are used for supervised learning within ANFIS.
- **Simulation Scenarios:** The controllers are evaluated under three operational conditions to assess robustness and adaptability:
  1. Nominal trajectory tracking (undisturbed conditions).
  2. Tracking under external input disturbances.
  3. Tracking under parameter variations affecting system dynamics.
- **Performance Evaluation:** The tracking performance of the FPID and FPIDANFIS controllers is assessed using the Integral of Time Absolute Error (ITAE) criterion. This metric provides a quantitative measure of tracking accuracy and control efficiency.
- **Comparative Analysis:** A comprehensive performance comparison between the proposed FPIDANFIS and baseline FPID is conducted based on ITAE values across different test conditions.

## 2 System modeling

**Fig 1:**
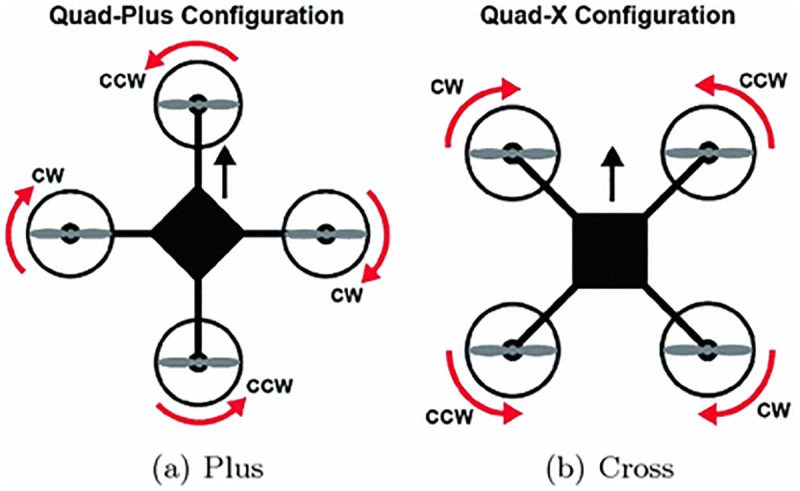
Quadrotor configurations and motion relative to propeller speed [57].

The modeling of a quadrotor system is a critical step in understanding its behavior and developing effective control strategies. Quadrotors, classified as unmanned aerial vehicles (UAVs) with four rotors, exhibit complex dynamics arising from the interaction of aerodynamics, mechanics, and control systems. This section outlines the fundamental concepts necessary for the system modeling of a quadrotor. This paper investigates the trajectory tracking control challenges associated with the ‘X’(cross) configuration quadrotor UAV. The propulsion system of this UAV consists of four motors, as illustrated in Fig 1, where adjustments in rotor speeds are utilized to control both attitude and position [57].

**Proposed Model:** This study adopts the ‘X’ (cross) configuration for the quadrotor model. The primary advantage of this configuration lies in its superior stability compared to the plus configuration, as the cross configuration utilizes two rotors to stabilize and guide the quadrotor’s movements [58,59].

**Fig 2:**
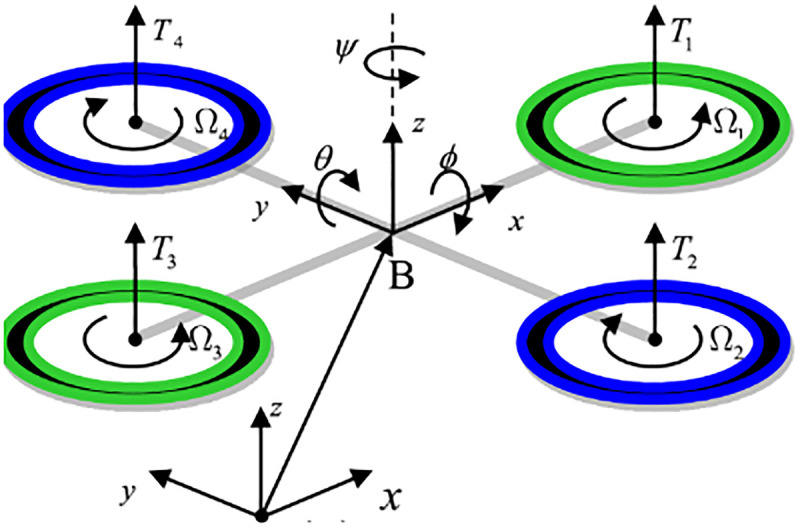
Reference frame [60].

The subsequent section focuses on Dynamics and Kinematics,of the quadrotor system. This analysis considers variables such as rotor thrust, torque, and the overall motion of the system in three-dimensional space. In the realm of the Rigid Body Control Model, we introduce how the quadrotor responds to external inputs and disturbances. This involves a comprehensive understanding of the forces and moments acting on the vehicle system. The subsequent segment encompasses the presentation of mathematical equations dictating the quadrotor’s motion. This includes translational and rotational dynamics, along with the presentation of equations of motion and their derivations from a reference frame in Fig 2. To precisely depict the position and attitude of the quadrotor UAV, the Earth-fixed coordinate system is utilized as ($o^{E}$,$x^{E}$,$y^{E}$,$z^{E}$) and the body coordinate system($o^{B},x^{B},y^{B},z^{B}$) are introduced.

The definition of the body structure and the coordinate system is shown in Fig 2. Modeling the quadrotor UAV is complex, to simplify the model establishment, the following assumptions are considered [58,59,60].

1. **Rigid and symmetrical structure:** This assumption implies that the quadrotor has a fixed shape and its mass is uniformly distributed.
2. **Center of gravity:** Assuming that the quadrotor’s center of gravity coincides with the body-fixed frame origin simplifies the analysis of the system dynamics. This assumption eliminates the need to consider the translation of the center of gravity.
3. **Rigid propellers:** Assuming that the propellers are rigid simplifies the modeling of the forces and moments generated by the propellers.
4. **Thrust and drag force:** The assumption that the thrust and drag forces are proportional to the square of the propeller’s speed.
5. **Motor dynamics:** Neglecting the motor’s resistance and inductance implies that the motor dynamics are significantly faster than the dynamics of the quadrotor itself. This assumption simplifies the modeling process by assuming that the motor instantaneously responds to changes in voltage and produces the corresponding output speed.

The orientation of the quadrotor is given by the rotation matrix *R* which depends on the three Euler angles (ϕ,θ,ψ).


$$\begin{array}{c} R(x,\phi )=\left[\begin{array}{ccc} 1 & 0 & 0\\ 0 & cos\phi & sin\phi \\ 0 & -sin\phi & cos\phi \end{array}\right] \end{array}$$ (1)

$$\begin{array}{c} R(y,\theta )=\left[\begin{array}{ccc} cos\theta & 0 & -sin\theta \\ 0 & 1 & 0\\ sin\theta & 0 & cos\theta \end{array}\right] \end{array}$$ (2)

$$\begin{array}{c} R(z,\psi )=\left[\begin{array}{ccc} cos\psi & sin\psi & 0\\ -sin\psi & cos\psi & 0\\ 0 & 0 & 1 \end{array}\right] \end{array}$$ (3)

$$\begin{array}{c} \left[\begin{array}{c} \frac{dx}{dt}\\ \frac{dy}{dt}\\ \frac{dz}{dt} \end{array}\right]={\;}_{B}^{E}R\left[\begin{array}{c} u\\ v\\ w \end{array}\right] \end{array}$$ (4)

**Transfer Matrix(**η)

$\zeta^{E}=[\phi ,\theta ,\psi {]}^{T}$ is in Inertial frame and Angular Velocity $\left(\Omega^{B}=[p,q,r{]}^{T}\right)$ is in Body frame.To relate the two, Transfer matrix(η) is used as [58,59,60]:


$$\begin{array}{c} {\dot{\zeta }}^{E}=\eta \Omega^{B} \end{array}$$ (5)

$$\begin{array}{c} \left[\begin{array}{c} \dot{\phi }\\ \dot{\theta }\\ \dot{\psi } \end{array}\right]=\eta \left[\begin{array}{c} p\\ q\\ r \end{array}\right] \end{array}$$ (6)

$$\begin{array}{c} \eta =\left[\begin{array}{ccc} 1 & \sin(\phi )(\tan(\theta ) & \cos(\phi )\tan(\theta )\\ 0 & \cos(\phi ) & -\sin(\phi )\\ 0 & \sin(\phi )\sec(\theta ) & \cos(\phi )\sec(\theta ) \end{array}\right] \end{array}$$ (7)

$$\begin{array}{c} \eta^{-1}=\left[\begin{array}{ccc} 1 & 0 & -\sin(\theta )\\ 0 & \cos(\phi ) & \sin(\phi )\cos(\theta )\\ 0 & -\sin(\phi ) & \cos(\phi )\cos(\theta ) \end{array}\right] \end{array}$$ (8)

**Newton-Euler Method formalism** The flight dynamics of the quadrotor UAV will be modeled using the Newton-Euler method [58,59,60,61]. The equations of motion, which combine the translational and rotational parameters which is described in Table 2 of the 6DOF system (consisting of 3 position and 3 angular orientation variables), are derived based on Euler’s two laws of motion.

**Table 2:** Parameters description [58].

Parameters	Description	Units
$\left[\begin{array}{ccc} x & y & z \end{array}\right]$	Linear position vector	m
$\left[\begin{array}{ccc} \phi & \theta & \psi \end{array}\right]$	Angular position vector	rad
$\left[\begin{array}{ccc} u & v & w \end{array}\right]$	Linear Velocity vector	m/s
$\left[\begin{array}{ccc} p & q & r \end{array}\right]$	Angular velocity vector	rad/s
$\left[\begin{array}{ccc} I_{xx} & I_{yy} & I_{zz} \end{array}\right]$	Moment of inertia vector	$kg\cdot m^{2}$
$F_{th}$	Total thrust generated by rotors	N
$\left[\begin{array}{ccc} \tau_{x} & \tau_{y} & \tau_{z} \end{array}\right]$	Control torques	N.m
*g*	Gravitational force	$m/s^{2}$
*m*	Total mass	kg
$\left[\begin{array}{cccc} \Omega_{1} & \Omega_{2} & \Omega_{3} & \Omega_{4} \end{array}\right]$	Rotors speeds vector	rad/s
*b*	Thrust coefficient	N.s $s^{2}$
*l*	Motor to center length	m
$\Omega_{r}$	residual rotor speed	rad/s
*d*	Drag coefficient	N.m.s.s
*Jr*	rotor inertia vector	$kg\cdot m^{2}$

The angular acceleration in the body frame is expressed as:


$$\begin{array}{c} \left\{ \begin{array}{c} \dot{p}=\frac{U_{2}}{I_{xx}}-\frac{qr(I_{yy}-I_{zz})}{I_{xx}}+\frac{Jr\Omega_{r}q}{I_{xx}}\\ \dot{q}=\frac{U_{3}}{I_{yy}}-\frac{rp(I_{zz}-I_{xx})}{I_{yy}}-\frac{Jr\Omega_{r}p}{I_{yy}}\\ \dot{r}=\frac{U_{4}}{I_{zz}}-\frac{pq(I_{xx}-I_{yy})}{I_{zz}} \end{array}\right. \end{array}$$ (9)

The quadrotor’s dynamics are described by four control inputs, represented as:


$$\begin{array}{c} 𝐔={\left[\begin{array}{cccc} U_{1} & U_{2} & U_{3} & U_{4} \end{array}\right]}^{T}, \end{array}$$

where:

- *U*_1_ represents the total thrust force (throttle) applied to the quadrotor body,
- *U*_2_ is the torque affecting the roll angle,
- *U*_3_ is the torque affecting the pitch angle, and
- *U*_4_ is the torque affecting the yaw (heading) angle.

The quadrotor’s translational and rotational dynamics are governed by the following equations [58, 62]:


$$\begin{array}{c} \left\{ \begin{array}{c} \ddot{x}=\left(\cos(\phi )\sin(\theta )\cos(\psi )+\sin(\phi )\sin(\psi )\right)\frac{U_{1}}{m}\\ \ddot{y}=\left(\cos(\phi )\sin(\theta )\sin(\psi )-\sin(\phi )\cos(\psi )\right)\frac{U_{1}}{m}\\ \ddot{z}=\left(\cos(\phi )\cos(\theta )\right)\frac{U_{1}}{m}-g\\ \ddot{\phi }=\frac{U_{2}}{I_{xx}}-\frac{\left(I_{xx}-I_{yy}\right)}{I_{xx}}\dot{\theta }\dot{\psi }+\frac{J_{r}\dot{\theta }\Omega_{r}}{I_{xx}}\\ \ddot{\theta }=\frac{U_{3}}{I_{yy}}-\frac{\left(I_{xx}-I_{zz}\right)}{I_{yy}}\dot{\phi }\dot{\psi }-\frac{J_{r}\dot{\phi }\Omega_{r}}{I_{yy}}\\ \ddot{\psi }=\frac{U_{4}}{I_{zz}}-\frac{\left(I_{yy}-I_{xx}\right)}{I_{zz}}\dot{\phi }\dot{\theta } \end{array}\right. \end{array}$$ (10)

The control inputs are used to calculate the rotor angular velocities, with their inverse relationship given as:


$$\begin{array}{c} \left\{ \begin{array}{c} \Omega_{1}=\sqrt{\frac{U_{1}}{4b}+\sqrt{2}\frac{U_{2}}{4bl}+\sqrt{2}\frac{U_{3}}{4bl}+\frac{U_{4}}{4d}}\\ \Omega_{2}=\sqrt{\frac{U_{1}}{4b}+\sqrt{2}\frac{U_{2}}{4bl}-\sqrt{2}\frac{U_{3}}{4bl}-\frac{U_{4}}{4d}}\\ \Omega_{3}=\sqrt{\frac{U_{1}}{4b}-\sqrt{2}\frac{U_{2}}{4bl}-\sqrt{2}\frac{U_{3}}{4bl}+\frac{U_{4}}{4d}}\\ \Omega_{4}=\sqrt{\frac{U_{1}}{4b}-\sqrt{2}\frac{U_{2}}{4bl}+\sqrt{2}\frac{U_{3}}{4bl}-\frac{U_{4}}{4d}} \end{array}\right. \end{array}$$ (11)

### 2.1 Virtual control

Virtual control is introduced to handle the underactuated nature of a quadrotor, which has six degrees of freedom (three translational and three rotational) but only four independent actuators. Because of this limitation, the vehicle cannot directly produce lateral motion; instead, horizontal movement is achieved indirectly by tilting the aircraft to generate the required thrust components.

To manage this coupling, the control system is typically divided into two layers. The outer loop (virtual control layer) computes the desired forces or accelerations required to reach a target position in the x, y, and z directions. These outputs are treated as virtual control inputs. The inner loop then converts these virtual commands into actual motor inputs by mapping them to individual rotor speeds using techniques such as control allocation or inverse kinematics. This structure simplifies the overall control problem and enables effective trajectory tracking in complex dynamics. The quadrotor is underactuated, with 4 control inputs and 6 states to be controlled. The states are indirectly controlled through the translational dynamics [58,59,60,63,62]:


$$\begin{array}{c} \left\{ \begin{array}{c} V_{x}=\ddot{x}=(\cos(\phi )\sin(\theta )\cos(\psi )+\sin(\phi )\sin(\psi ))\frac{U_{1}}{m}\\ V_{y}=\ddot{y}=(\cos(\phi )\sin(\theta )\sin(\psi )-\sin(\phi )\cos(\psi ))\frac{U_{1}}{m}\\ V_{z}=\ddot{z}=(\cos(\phi )\cos(\theta ))\frac{U_{1}}{m}-g \end{array}\right. \end{array}$$ (12)

where $V_{x}$, $V_{y}$, and $V_{z}$ are the virtual control inputs.

Based on these equations, the lift force (altitude control signal) and the desired Euler angles are calculated as [58]:


$$\begin{array}{c} \left\{ \begin{array}{c} U_{1}=m\sqrt{V_{x}^{2}+V_{y}^{2}+{\left(V_{z}+g\right)}^{2}}\\ \theta_{r}=\arctan\left(\frac{V_{x}\cos(\psi_{r})+V_{y}\sin(\psi_{r})}{V_{z}+g}\right)\\ \phi_{r}=\arctan\left(\frac{\cos(\theta_{r})\left(V_{x}\sin(\psi_{r})-V_{y}\cos(\psi_{r})\right)}{V_{z}+g}\right) \end{array}\right. \end{array}$$ (13)

## 3 Problem statement

This paper addresses the trajectory tracking control problem of a quadrotor UAV, as described in Equation (10), with the goal of ensuring precise tracking of a predefined trajectory by minimizing the tracking error *e*(*t*), defined in Equation (26).

To achieve this objective, an intelligent and adaptive control strategy is required. A Gaussian membership function, given in Equation (18), is employed to enhance control adaptability, while a hybrid learning algorithm is utilized to optimize the error gradient. This optimization process generates the optimal intelligent adaptive control signal *U*, formulated in Equation (25).

The following sections provide a detailed explanation of the ANFIS architecture and its learning mechanism, which are designed to minimize the tracking error and improve the overall trajectory tracking performance.

### 3.1 ANFIS architecture and learning algorithm

**ANFIS Structure** To illustrate the ANFIS architecture, two fuzzy IF-THEN rules based on a first-order Sugeno model are considered:


$$\begin{array}{cc} \text{Rule 1}: & \;\text{IF }e(t)\text{ is }A_{1}\text{ AND }\\ \frac{de(t)}{dt}\text{ is }B_{1},\\ \text{ THEN }f_{1}=p_{1}e(t)+q_{1}\frac{de(t)}{dt}+r_{1} \end{array}$$ (14)

$$\begin{array}{cc} \text{Rule 2}: & \;\text{IF }e(t)\text{ is }A_{2}\text{ AND }\\ \frac{de(t)}{dt}\text{ is }B_{2},\\ \text{ THEN }f_{2}=p_{2}e(t)+q_{2}\frac{de(t)}{dt}+r_{2} \end{array}$$ (15)

where:

- *e*(*t*) and $\frac{de(t)}{dt}$ are the input variables,
- $A_{i}$ and $B_{i}$ are the fuzzy sets,
- $f_{i}$ are the outputs within the fuzzy region specified by the fuzzy rule, and
- $p_{i}$, $q_{i}$, and $r_{i}$ are design parameters determined during the training process.

**Fig 3:**
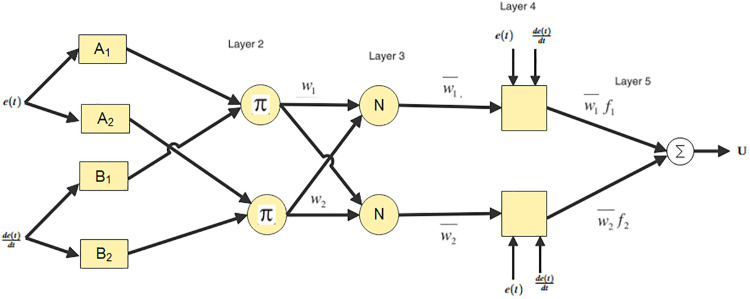
ANFIS architecture.

The reasoning mechanism for this Sugeno model, which forms the basis of the ANFIS model, is illustrated in Fig 3. The ANFIS architecture consists of five layers, where circles represent fixed nodes and squares indicate adaptive nodes. Each layer performs a distinct function in processing the input data.

### 3.2 Layer descriptions

**Layer 1: Fuzzification Layer** All nodes in this layer are adaptive and compute the membership grade of the inputs using fuzzy membership functions. The outputs are given by:


$$\begin{array}{c} O_{1,i}=A_{i}(e(t)),\;i=1,2, \end{array}$$ (16)

$$\begin{array}{c} O_{1,i}=B_{i-2}(\frac{d}{e(t)}),\;i=3,4. \end{array}$$ (17)

where $A_{i}(e(t))$ and $B_{i}(\frac{de(t)}{dt})$ represent the fuzzy membership functions associated with linguistic labels (e.g., high, low). These functions can adopt the Gaussian membership function:


$$\begin{array}{c} A_{i}(e(t))=\exp\left(-{\left(\frac{e(t)-c_{i}}{a_{i}}\right)}^{2}\right), \end{array}$$ (18)

where $a_{i}$, $b_{i}$, and $c_{i}$ are parameters that define the shape of the membership function.

**Layer 2: Rule Firing Strength Computation** This layer consists of fixed nodes that compute the firing strength of each rule using the AND operator:


$$\begin{array}{c} O_{2,i}=w_{i}=A_{i}(e(t))\cdot B_{i}(\frac{de(t)}{dt}),\;i=1,2. \end{array}$$ (19)

**Layer 3: Normalization Layer** The nodes in this layer normalize the firing strengths from the previous layer:


$$\begin{array}{c} O_{3,i}={\bar{w}}_{i}=\frac{w_{i}}{w_{1}+w_{2}},\;i=1,2. \end{array}$$ (20)

These normalized values, ${\bar{w}}_{i}$, are known as normalized firing strengths.

**Layer 4: Defuzzification Layer** The nodes in this layer are adaptive, computing the contribution of each rule to the final output:


$$\begin{array}{c} O_{4,i}={\bar{w}}_{i}f_{i}={\bar{w}}_{i}(p_{i}e(t)+q_{i}(\frac{de(t)}{dt})+r_{i}),\;i=1,2. \end{array}$$ (21)

where ${\bar{w}}_{i}$ represents the normalized firing strengths, and $p_{i}$, $q_{i}$, and $r_{i}$ are the consequent parameters.

**Layer 5: Output Layer** This layer consists of a single fixed node that performs the summation of all incoming signals to compute the final output:


$$\begin{array}{c} O_{5}=\sum_{i}{\bar{w}}_{i}f_{i}=\frac{\sum_{i}w_{i}f_{i}}{\sum_{i}w_{i}}. \end{array}$$ (22)

### 3.3 Hybrid learning algorithm

The ANFIS learning process employs a hybrid algorithm combining the least squares method and gradient descent to optimize both premise and consequent parameters. The training process consists of two passes:

- **Forward Pass:** The node outputs propagate forward through the network until Layer 4, where the consequent parameters $\left(p_{i},q_{i},r_{i}\right)$ are updated using the least squares method.
- **Backward Pass:** The error signals propagate backward through the network, and the premise parameters $\left(a_{i},b_{i},c_{i}\right)$ are updated using the gradient descent method.

The hybrid learning approach improves convergence speed by reducing the search space dimensions compared to conventional backpropagation methods [64]. The overall output of the ANFIS model can be expressed as:


$$\begin{array}{c} U={\bar{w}}_{1}f_{1}+{\bar{w}}_{2}f_{2}, \end{array}$$ (23)

which, when expanded, is given by:


$$\begin{array}{c} U={\bar{w}}_{1}(p_{1}e(t)+q_{1}(\frac{de(t)}{dt})+r_{1})+{\bar{w}}_{2}(p_{2}e(t)+q_{2}(\frac{de(t)}{dt})+r_{2}). \end{array}$$ (24)

Rewriting in terms of individual components:


$$\begin{array}{c} U=e(t)\sum_{i=1}^{2}{\bar{w}}_{i}p_{i}+\dot{e}(t)\sum_{i=1}^{2}{\bar{w}}_{i}q_{i}+\sum_{i=1}^{2}{\bar{w}}_{i}r_{i} \end{array}$$ (25)

The least squares method is employed to determine optimal values for the linear consequent parameters $\left(p_{i},q_{i},r_{i}\right)$. When the premise parameters are not fixed, the search space increases, leading to slower convergence. The hybrid approach mitigates this issue by separately optimizing premise and consequent parameters.

The effectiveness of the hybrid algorithm has been demonstrated in improving ANFIS training efficiency by balancing parameter optimization through complementary learning strategies [65,64].

## 4 Controller design

**Fig 4:**
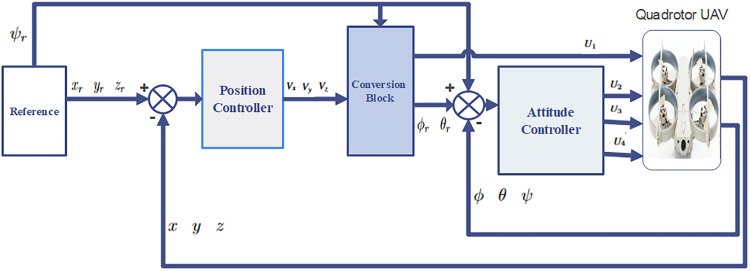
Control scheme block diagram [58].

This section details the design of Fuzzy PID (FPID) and Fuzzy PID-based Adaptive Neuro-Fuzzy Inference System (FPIDANFIS) controllers. The FPID controller combines the conventional PID structure with fuzzy logic to enable real-time adaptive gain adjustment, improving performance in nonlinear systems. Conversely, the FPIDANFIS controller employs a data-driven approach, learning from system behavior to optimize control actions for complex systems such as quadrotors.Gaussian membership functions (MFs) are widely used in fuzzy logic systems and Adaptive Neuro-Fuzzy Inference Systems (ANFIS) due to their smooth curves, differentiable nature, and their ability to closely represent real-world continuous processes. Although simpler shapes such as triangular and trapezoidal MFs require less computation, Gaussian functions are preferred when higher accuracy, smooth transitions, and strong adaptability in modeling are required. To enhance trajectory tracking, the control deviation at each instant is correlated with the system’s historical state. The generalized control scheme block diagram is shown in Fig 4.

The error and its derivative are defined as:


$$\begin{array}{c} \left\{ \begin{array}{c} e(t)=r(t)-y(t)\\ de(t)=\frac{de(t)}{dt} \end{array}\right. \end{array}$$ (26)

where $r={\left[\begin{array}{cccccc} \phi_{r} & \theta_{r} & \psi_{r} & x_{r} & y_{r} & z_{r} \end{array}\right]}^{T}$ denotes the desired reference signal, and $y={\left[\begin{array}{cccccc} \phi & \theta & \psi & x & y & z \end{array}\right]}^{T}$ represents the output states of the quadrotor.

### 4.1 Fuzzy PID controller design (FPID)

The FPID controller uses a fuzzy system for online PID gain tuning [58,66,67,68]. This is essential due to the nonlinear and underactuated nature of quadrotors, making classical PID control inadequate. The design involves the following steps:

1. **Define Tuning Rules:**Establish fuzzy IF-THEN rules based on system requirements to determine PID gain adjustments in response to various inputs.
2. **Create a Fuzzy System:** Combine the rules into a fuzzy system capable of real-time PID tuning based on feedback signals from the quadrotor.

**Fig 5:**
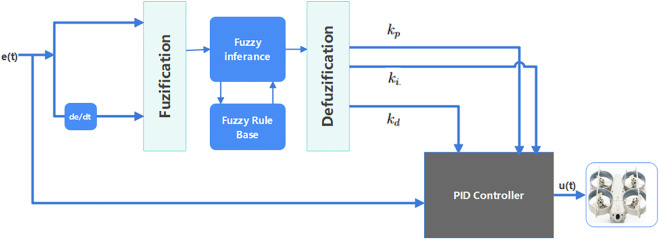
Structure of fuzzy PID controller [58].

**Fig 6:**
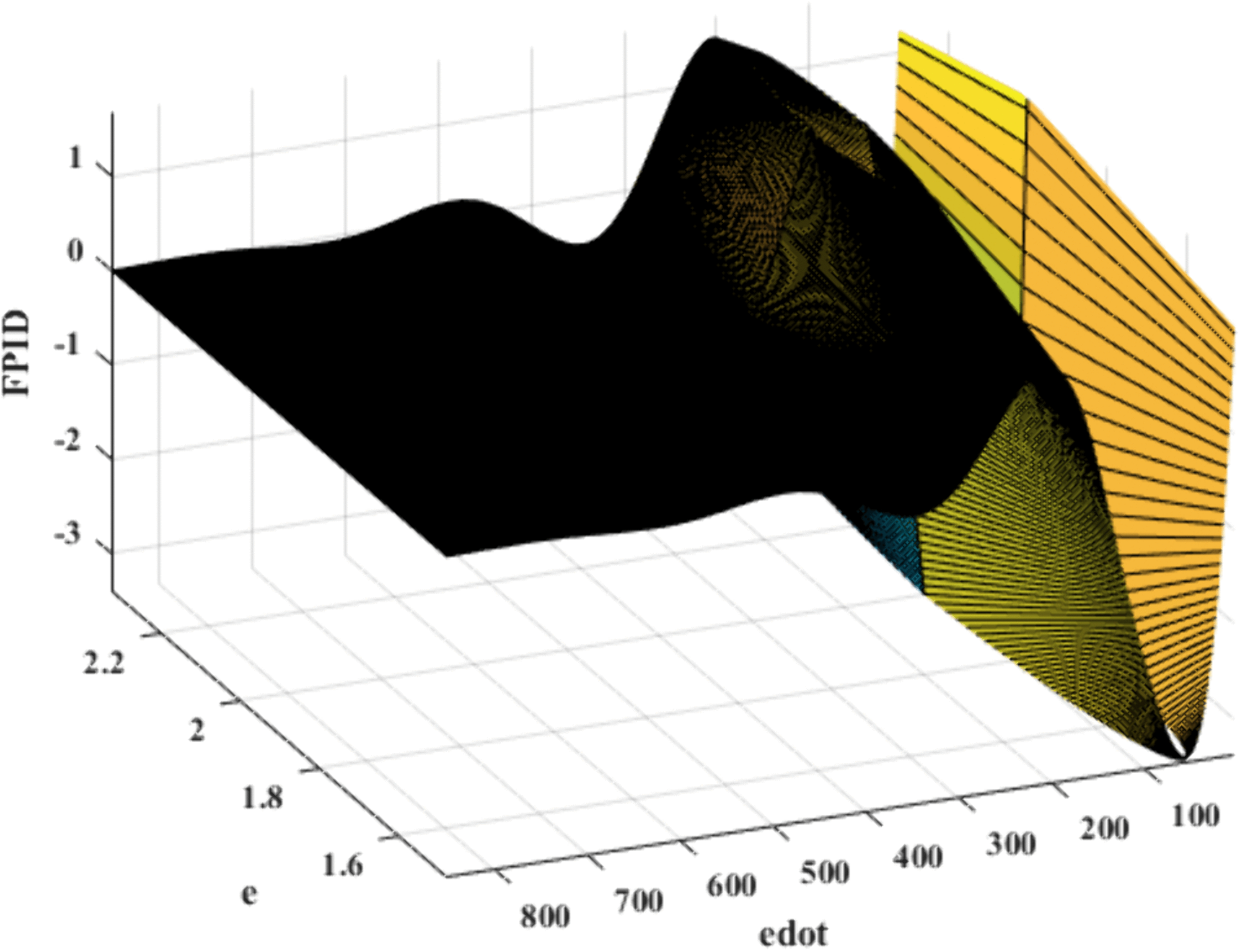
Surface plot input/output data of FPID controller.

The structure of the FPID controller is shown in Fig 5 and its input/output surface plot is depicted in Fig 6.

The fuzzy inference system uses a set of linguistic rules to determine the parameters $K_{p}$, $K_{i}$, and $K_{d}$ automatically [58,69,70,68]. Input variables use seven fuzzy labels (NB: Negative Big, NM: Negative Medium, NS: Negative Small, ZO: Zero, PS: Positive Small, PM: Positive Medium, PB: Positive Big), while output variables use seven labels (VVS: Very Very Small, VS: Very Small, S: Small, M: Medium, B: Big, VB: Very Big, VVB: Very Very Big). These are detailed in Tables 3 and 4.

**Table 3:** Fuzzy rules for *K_d_* [58,68].

de\e	NB	NM	NS	Z	PS	PM	PB
NB	M	B	VB	VVB	VB	B	M
NM	S	M	B	VB	B	M	S
NS	VS	S	M	B	M	S	VS
Z	VVS	VS	S	M	S	VS	VVS
PS	VS	S	M	B	M	S	VS
PM	S	M	B	VB	B	M	S
PB	M	B	VB	VVB	VB	B	M

**Table 4:** Fuzzy rules for $K_{p}$ and *K_i_* [58,68].

de\e	NB	NM	NS	Z	PS	PM	PB
NB	M	S	VS	VVS	VS	S	M
NM	B	M	S	VS	S	M	B
NS	VB	B	M	S	M	B	VB
Z	VVB	VB	B	M	B	VB	VVB
PS	VB	B	M	S	M	B	VB
PM	B	M	S	VS	S	M	B
PB	M	S	VS	VVS	VS	S	M

For position and attitude control, the error is normalized to [−1,1], the error rate to [−10,10], and the output ranges are [0.2, 0.7], [0.001, 0.01], and [0.1, 0.15] for proportional, integral, and derivative gains, respectively. The system employs product-sum inference and center-of-gravity defuzzification [58,66,68,71,72].

The fuzzy controller output is:


$$\begin{array}{c} U_{\text{FPID}}(t)=k_{p}e(t)+k_{i}\int_{0}^{t}e(t)dt+k_{d}\frac{de(t)}{dt} \end{array}$$ (27)

### 4.2 A novel fuzzy PID based adaptive neuro-fuzzy inference system (FPIDANFIS) controller

**Fig 7:**
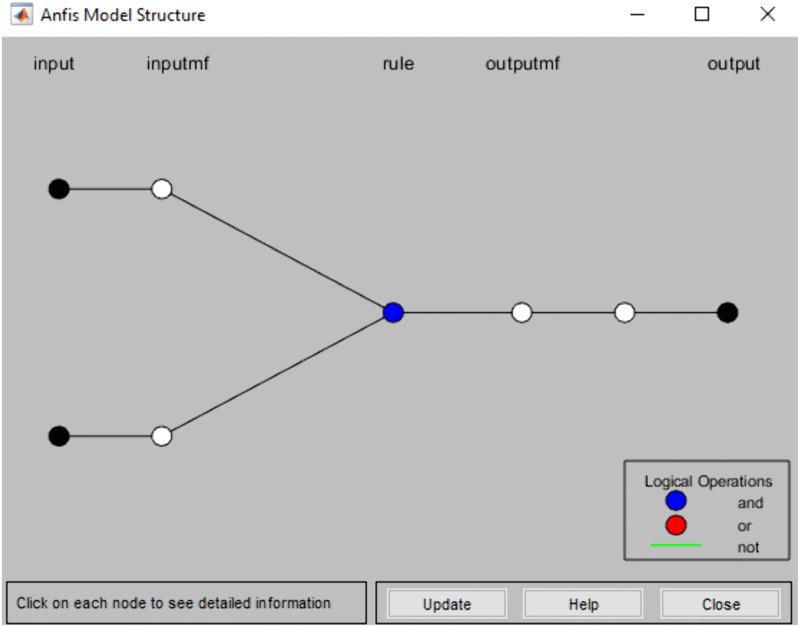
ANFIS model structure.

**Fig 8:**
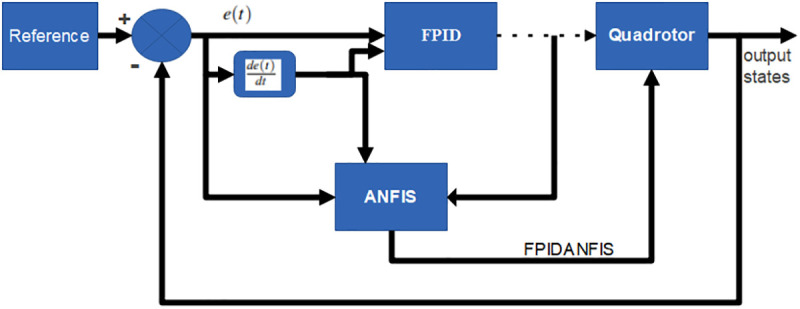
A novel FPIDANFIS control scheme.

**Fig 9:**
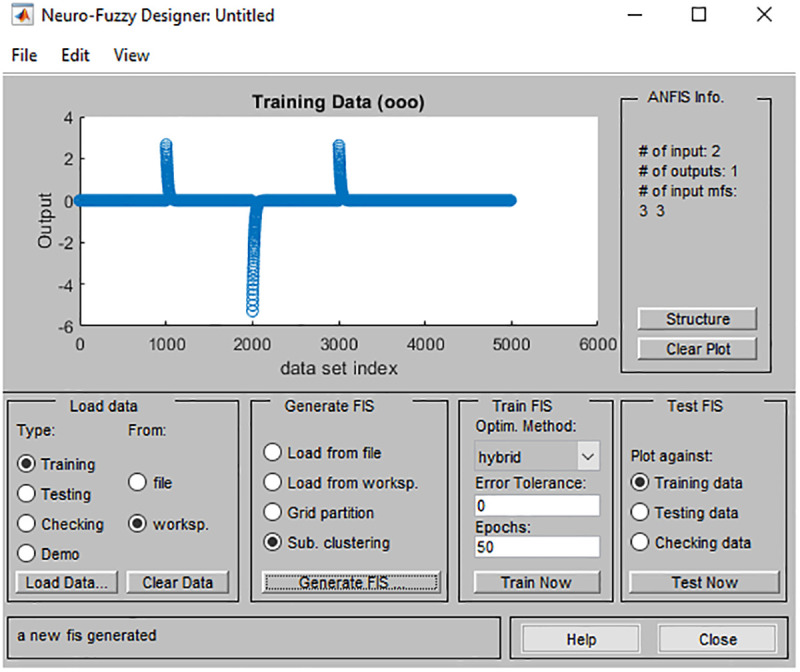
Training data interface using neuro-fuzzy designer toolbox.

**Fig 10:**
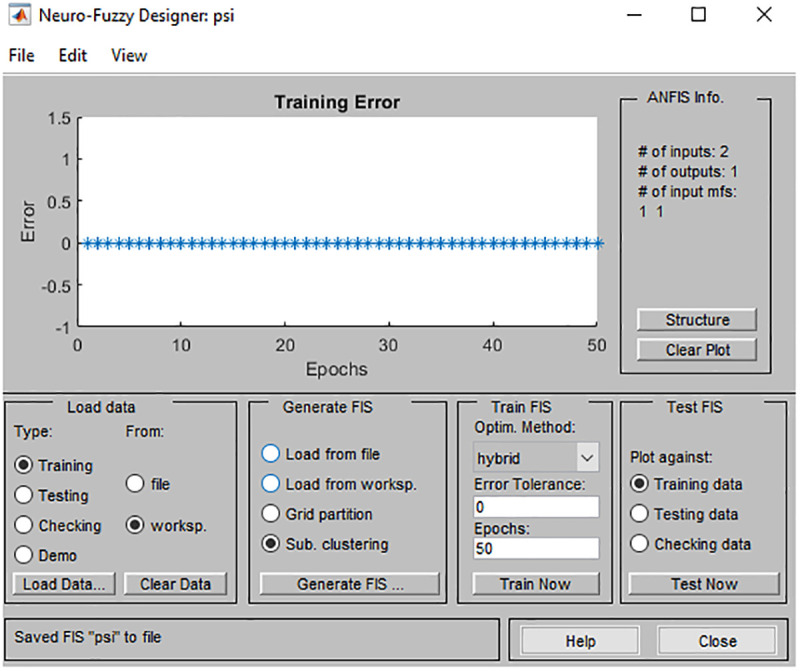
ANFIS training using neuro-fuzzy designer toolbox.

**Fig 11:**
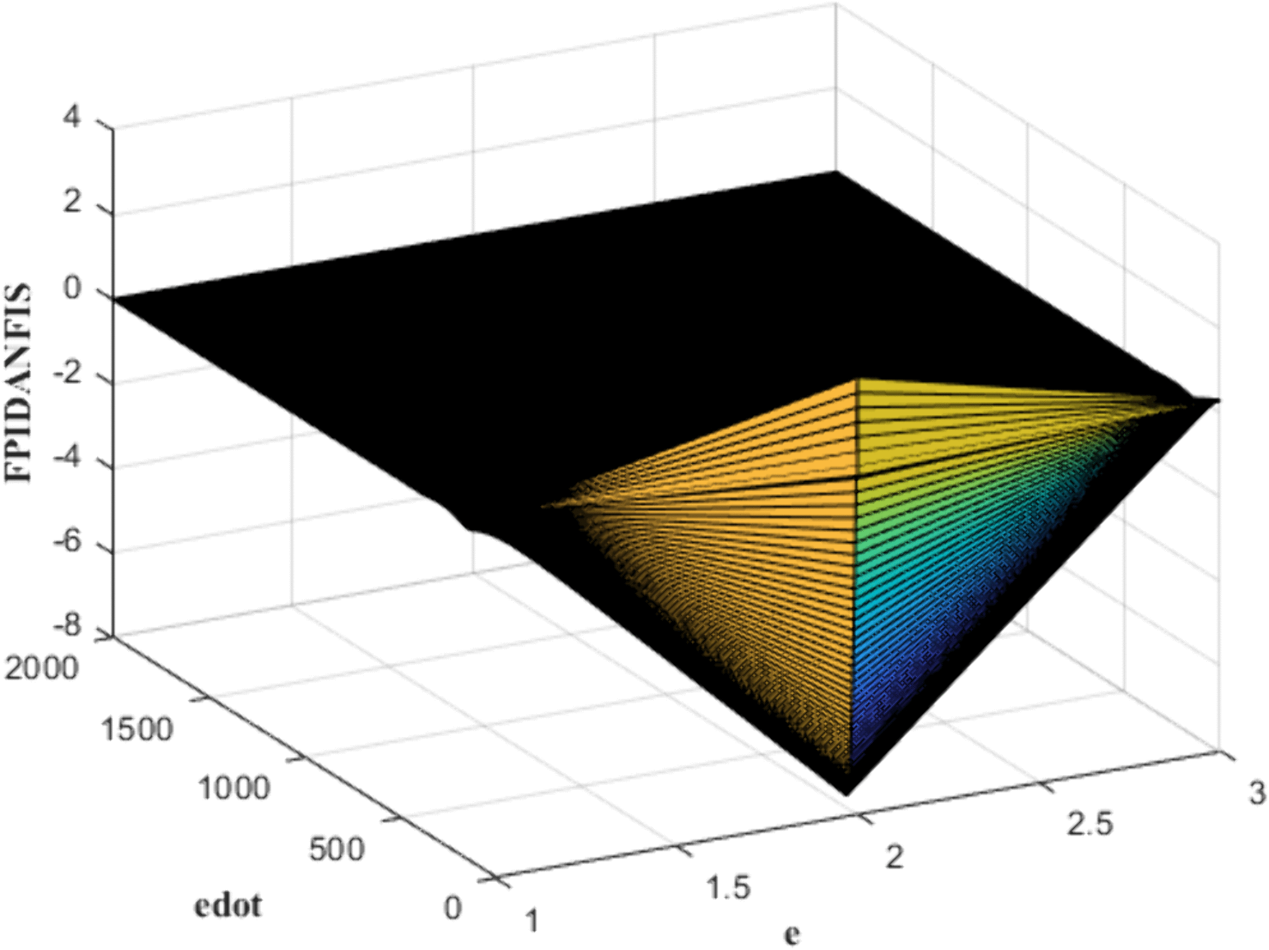
Surface plot input/output data of FPIDANFIS controller.

**Fig 12:**
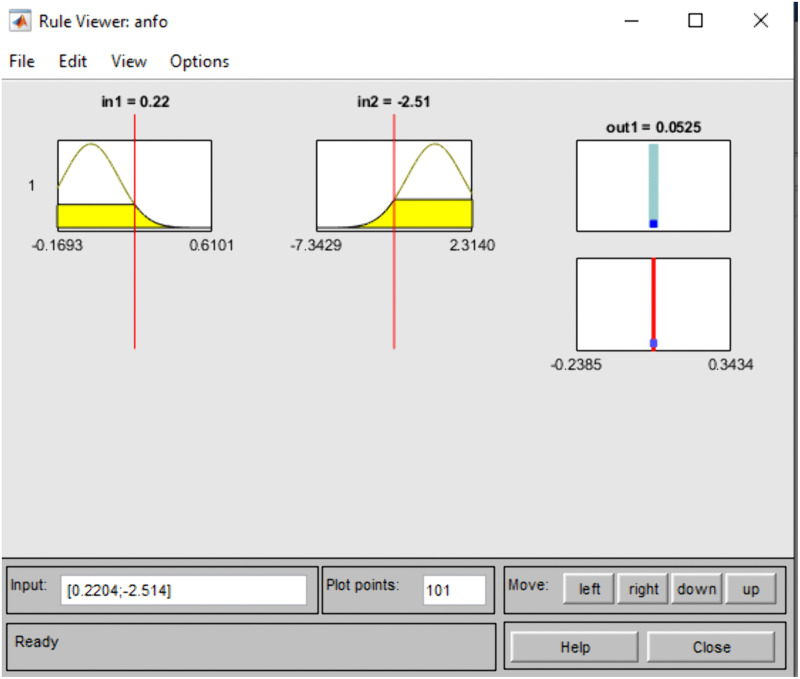
Fuzzy rule for FPIDANFIS controller.

**Fig 13:**
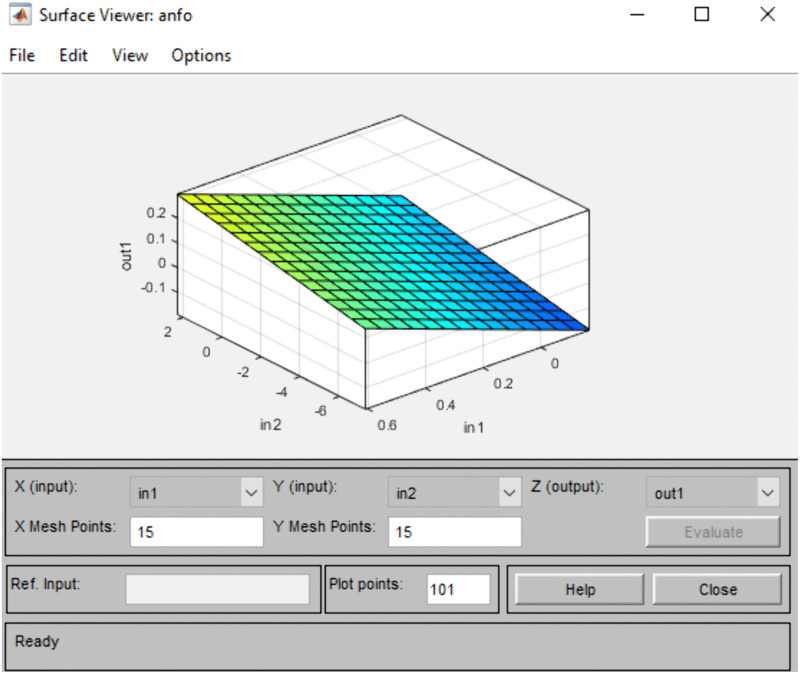
Surface plot of FPIDANFIS controller.

Adaptive Neuro-Fuzzy Inference Systems (ANFIS) integrate the learning capabilities of neural networks with the imprecision-handling strengths of fuzzy logic. This hybrid approach effectively models complex nonlinear relationships and adapts to uncertainties within data, making ANFIS a robust tool for outcome estimation and system control. A major limitation of fuzzy or neuro-fuzzy systems is their failure to deal with high-dimensional datasets [73]. The Adaptive Neuro-Fuzzy Inference System (ANFIS) serves as a classifier by combining fuzzy logic with neural networks in a hybrid approach. It implements a fuzzy inference system (FIS) within an adaptive fuzzy neural network framework, leveraging the explicit knowledge representation of FIS and the learning capabilities of artificial neural networks [74].Adaptive Neuro-Fuzzy Inference System (ANFIS) in prediction using data sets and fusion of fuzzy logic and neural network with a hybrid learning algorithm can be very useful in any research problem beyond engineering research even in social science research like Psychological research [75]. The Adaptive Neuro-Fuzzy Inference System (ANFIS) controller effectively addresses control challenges in uncertain systems by combining the fuzzy system’s ability to handle uncertainty with the self-learning capability of neural networks for parameter optimization [76]. For nonlinear and uncertain ship motion, an ANFIS-based course-keeping controller is designed to simplify parameter adjustment and enhance control performance [77].The Adaptive Neuro-Fuzzy Inference System (ANFIS) utilizes error and its derivative as inputs, along with the output of the fuzzy PID (FPID) controller. For this study, a dataset comprising 5001 samples, including two inputs (error and its derivative) and one output (FPID controller), is generated in the MATLAB workspace. Offline training is performed using the Neuro-Fuzzy Designer toolbox, employing a hybrid learning algorithm to optimize the system, and the gaussian membership function is used and the output is constant. The model structure is in Fig 7 and the proposed control strategy is depicted in Fig 8 whereas, the training data interface, ANFIS training using Neuro-Fuzzy Designer toolbox and its input output surface plots illustrated in Figs 9, 10, and 11 respectively whereas, the fuzzy rule view and surface plot of the FPIDANFIS controller is dipicted in Figs 12 and 13 respectively.

Table 5 presents the ANFIS training configuration and parameter settings employed in the MATLAB environment. The table specifies the number and categories of membership functions assigned to the input variables, which are crucial for achieving accurate system representation. It also summarizes the training parameters, including the maximum number of epochs, the optimization algorithm adopted (e.g., hybrid learning or backpropagation), and the learning rate settings used throughout the training process to enhance convergence and model performance.

**Table 5:** ANFIS model structure and training configuration.

Parameter	Value
ANFIS Type	Sugeno Fuzzy Inference System
Number of Input Variables	2
Number of Output Variables	1
Number of Input Membership Functions	2 (1 per input)
Number of Output Membership Functions	1
Number of Fuzzy Rules	1
Rule Connection Operator	AND
Consequent Type	Linear
FIS Structure Generation	ANFIS Editor (MATLAB)
Training Method	Hybrid Learning Algorithm
Maximum Training Epochs	100
Initial Step Size	0.01
Step Size Increase Rate	1.10
Step Size Decrease Rate	0.90
Error Tolerance	0
Learning Rate	0.01
Software Environment	MATLAB ANFIS Toolbox
Objective	Nonlinear Mapping and Adaptive Learning

## 5 Simulation

The quadrotor simulation incorporates the parameters listed in Table 6 within the MATLAB environment. The performance of the proposed FPIDANFIS controller is evaluated for trajectory tracking and compared to the standard FPID controller under varying conditions. Simulations are performed using MATLAB 2023a Simulink, and the results demonstrate the superior effectiveness of the FPIDANFIS controller in achieving accurate helical trajectory tracking.The reference signal for helical trajectory tracking is defined as:


$$\begin{array}{c} \left\{ \begin{array}{c} x_{r}(t)=\cos(1.05t)\\ y_{r}(t)=\sin(1.05t)\\ z_{r}(t)=3+\frac{t}{4}\\ \psi_{r}(t)=\frac{\pi }{4} \end{array}\right. \end{array}$$ (28)

**Table 6:** Parameter values [58].

Parameter	Value
$I_{xx}$	$8.5532\times 10^{-3}$
$I_{yy}$	$8.5532\times 10^{-3}$
$I_{zz}$	$1.476\times 10^{-2}$
*g*	$9.81\times 10^{0}$
*m*	$1\times 10^{0}$
*b*	$7.66\times 10^{-5}$
*d*	$5.63\times 10^{-6}$
*l*	$2.2\times 10^{-1}$
$J_{r}$	$0.1\times 10^{-3}$

**Fig 14:**
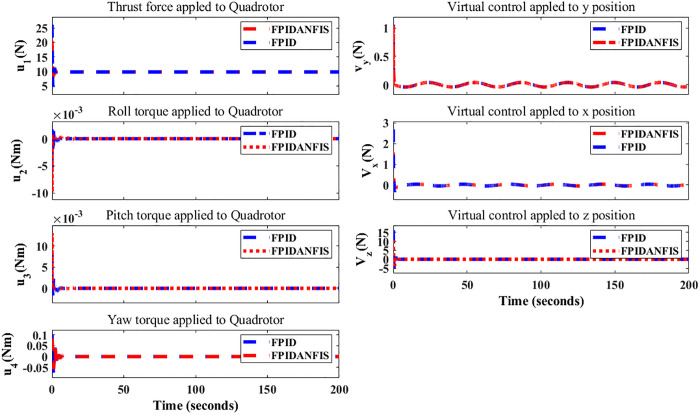
Control effort for helical trajectory tracking.

**Fig 15:**
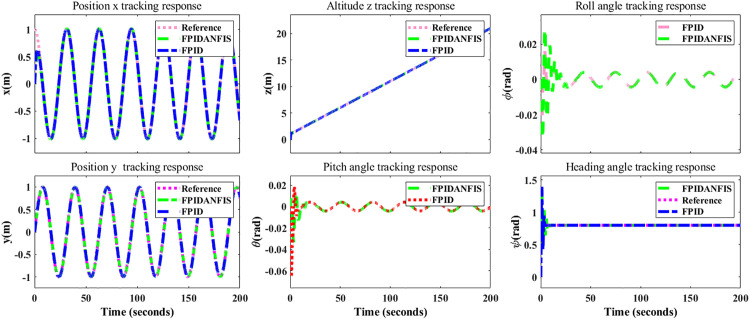
Position tracking response for helical trajectory tracking.

**Fig 16:**
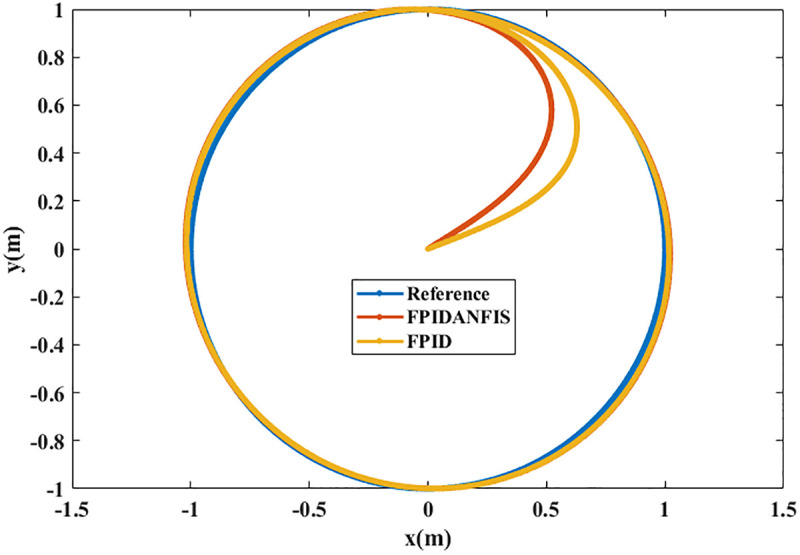
xy tracking response for helical trajectory tracking.

**Fig 17:**
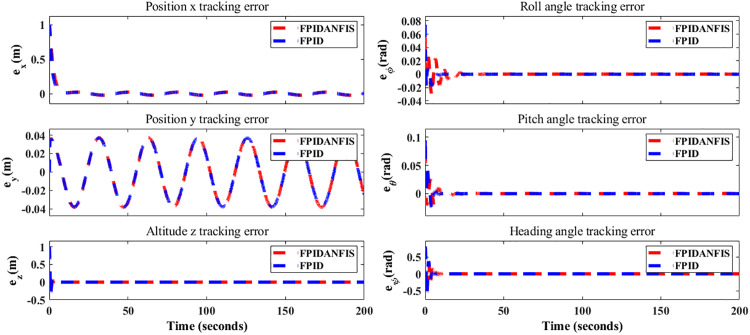
Tracking error for helical trajectory tracking.

**Fig 18:**
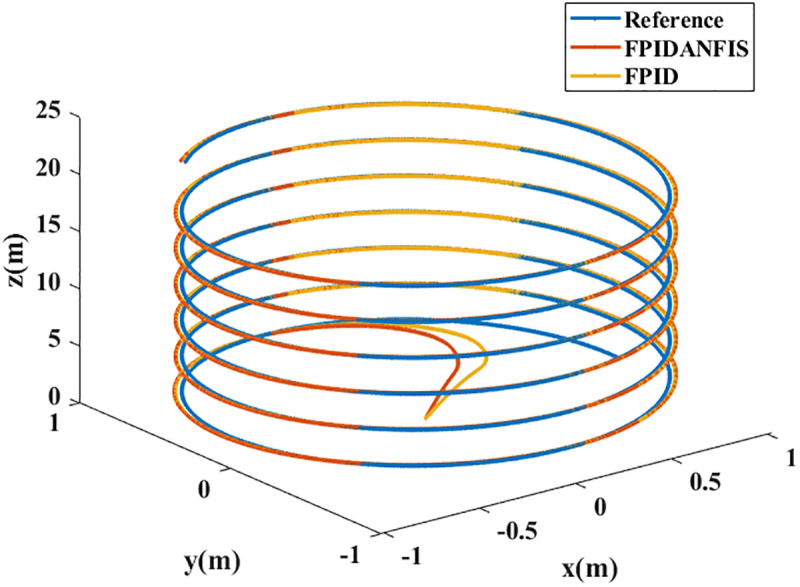
3D plot for helical trajectory tracking.

The controllers’ (FPIDANFIS and FPID) tracking performance under undisturbed conditions. The results for helical trajectory tracking are shown in Figs 14–18.

The Table 7 presents a comparison of the computational time required by the FPIDANFIS and FPID controllers for helical trajectory tracking. It can be observed that the FPIDANFIS controller exhibits a lower computational time of 6.2367549 seconds compared to the FPID controller, which requires 7.46578 seconds. This indicates that the FPIDANFIS approach is computationally more efficient, achieving faster execution while maintaining the control functionality.

**Table 7:** Computational time comparison of FPIDANFIS and FPID controllers.

Controller	Elapsed Time (s)
FPIDANFIS	6.2367549
FPID	7.46578

For infinity trajectory tracking the reference signal is:


$$\begin{array}{c} \left\{ \begin{array}{c} x_{r}(t)=1+\sin\left(2t\right)\\ y_{r}(t)=1-\cos\left(t\right)\\ z_{r}(t)=2\\ \psi_{r}(t)=0 \end{array}\right. \end{array}$$ (29)

**Fig 19:**
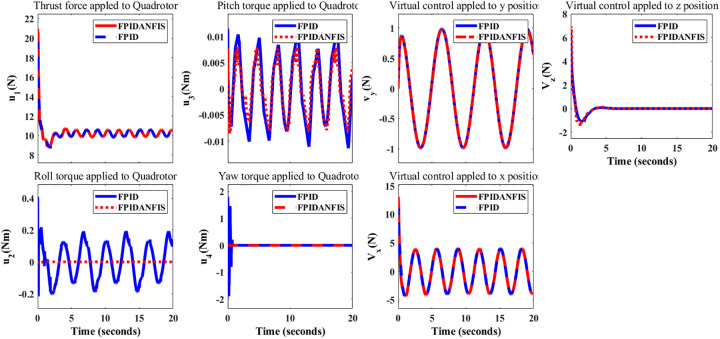
Control effort for infinity trajectory tracking.

**Fig 20:**
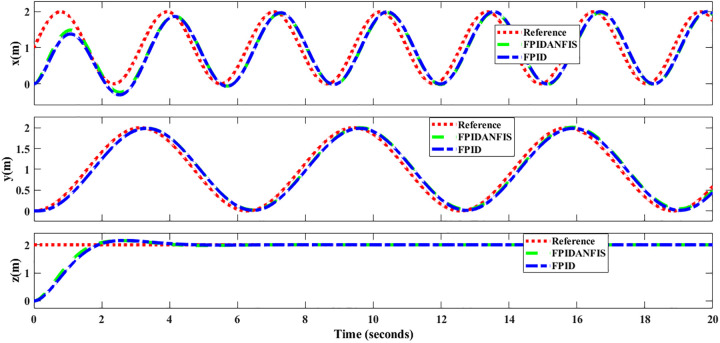
Position tracking response for infinity trajectory tracking.

**Fig 21:**
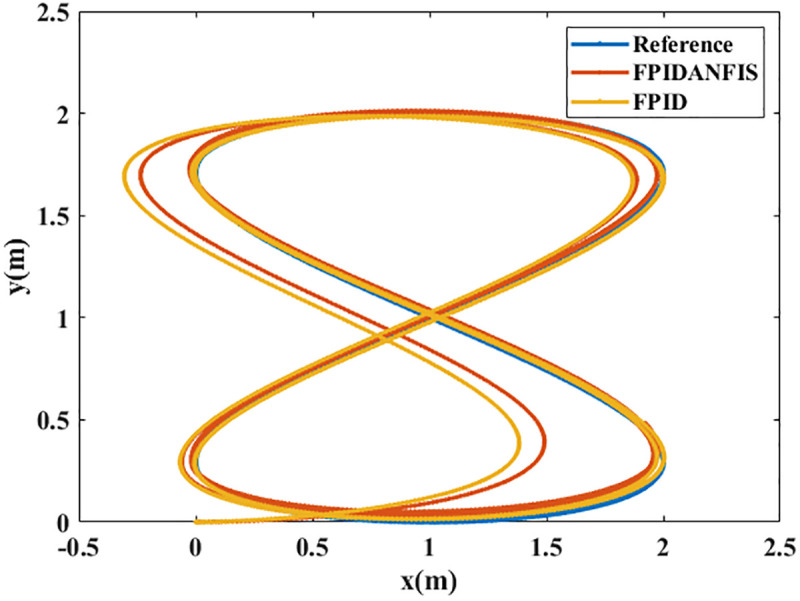
xy tracking response for infinity trajectory tracking.

**Fig 22:**
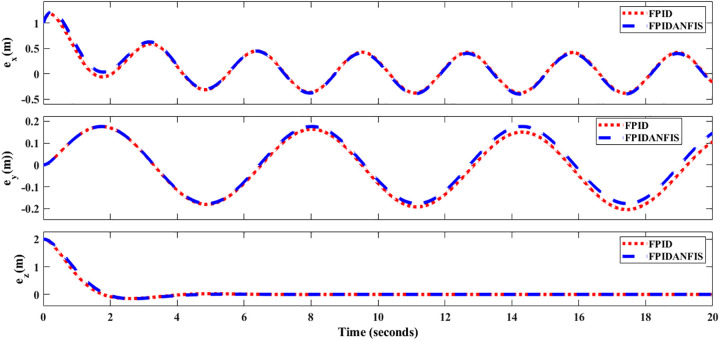
Tracking error for infinity trajectory tracking.

**Fig 23:**
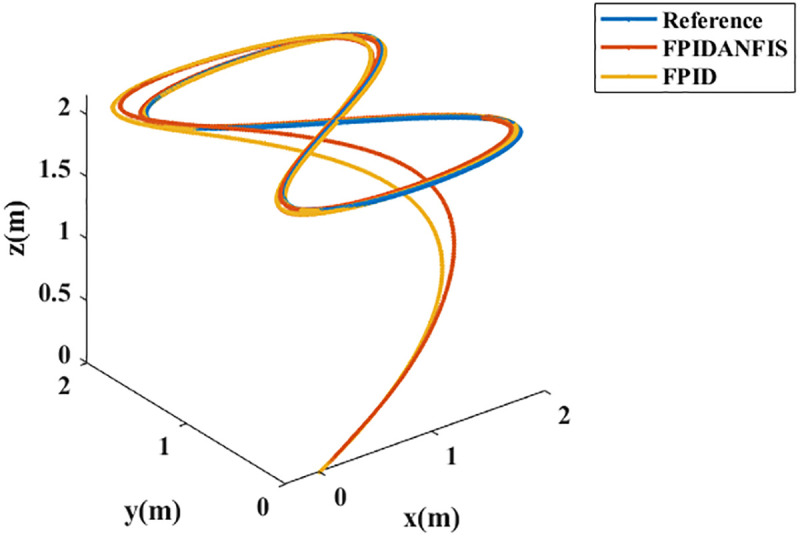
3D plot for infinity trajectory tracking.

The controllers’ (FPIDANFIS and FPID) tracking performance under undisturbed conditions. The results for infinity trajectory tracking are shown in Figs 19, 20, 21, 22 and 23.

**Algorithm 1: Pseudocode for triangular trajectory**

1: **if** *t* < 10

2: $\;x_{r}\leftarrow 0.5t$

3: $\;y_{r}\leftarrow 0$

4: $\;z_{r}\leftarrow 5$

5: **else if** 10≤t≤20

6: $\;x_{r}\leftarrow -0.25t+7.5$

7: $\;y_{r}\leftarrow 0.5t-5$

8: $\;z_{r}\leftarrow 5$

9: **else if** 20<t≤30

10: $\;x_{r}\leftarrow -0.25t+7.5$

11: $\;y_{r}\leftarrow -0.5t+15$

12: $\;z_{r}\leftarrow 5$

13: **else**

14: $\;x_{r}\leftarrow 0$

15: $\;y_{r}\leftarrow 0$

16: $\;z_{r}\leftarrow 5$

17: **end if**

**Fig 24:**
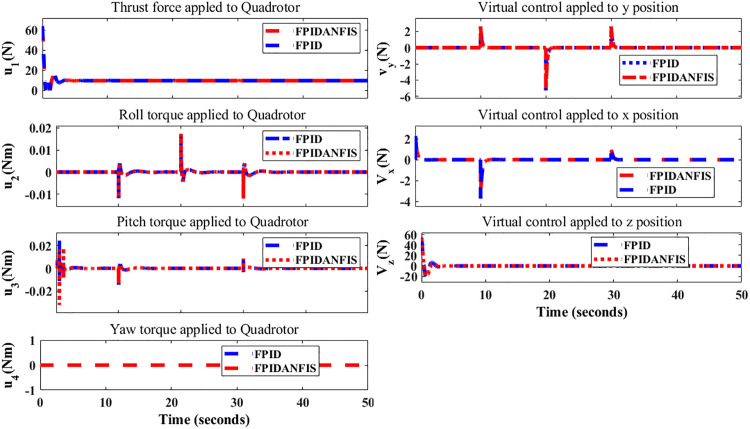
Control effort for triangular trajectory tracking.

**Fig 25:**
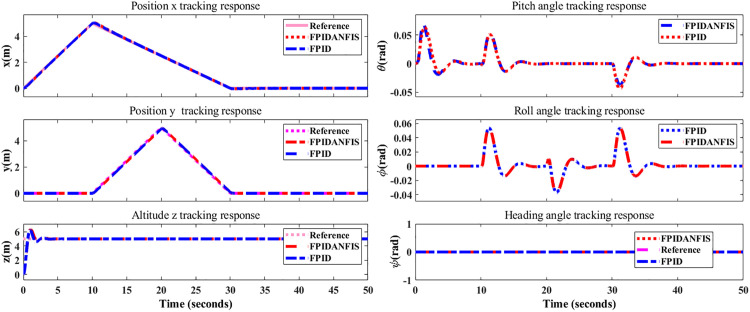
Position tracking response for triangular trajectory tracking.

**Fig 26:**
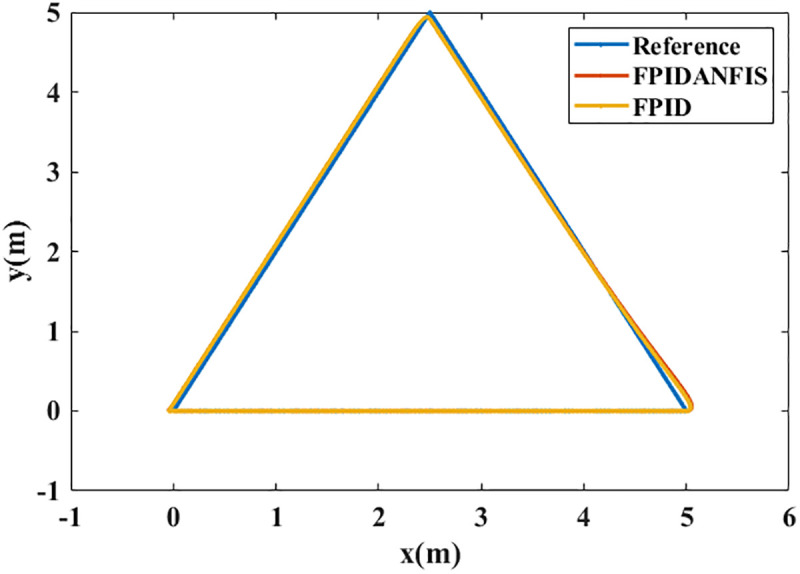
xy tracking response for triangular trajectory tracking.

**Fig 27:**
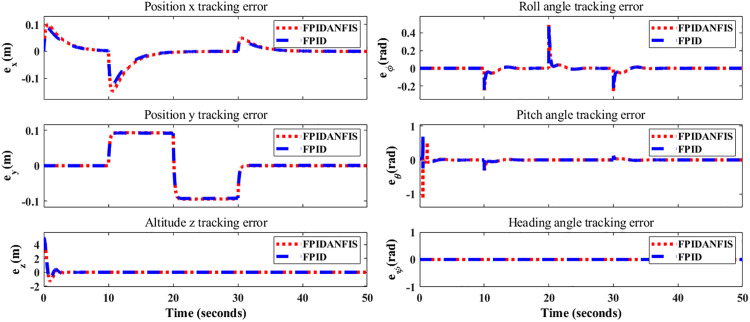
Tracking error for triangular trajectory tracking.

**Fig 28:**
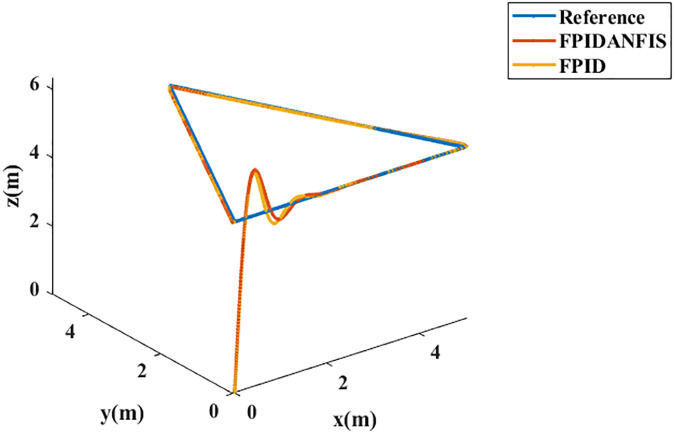
3D plot for triangular trajectory tracking.

The controllers’ (FPIDANFIS and FPID) tracking performance under undisturbed conditions. The results for triangular trajectory tracking are shown in Figs 24, 25, 26, 27 and 28.

## 6 Disturbance rejection capability and robustness to parameter variation

This section assesses the disturbance rejection capability and robustness check with the FPIDANFIS and FPID controllers under conditions of parameter variation and input disturbance. To replicate real-world scenarios, a random force representing wind disturbances was introduced as an external input, affecting the *x*, *y*, and *z* positions of the system. Additionally, parameter variations were modeled by increasing the total quadrotor mass by 20% and the inertia values along the *x*, *y*, and *z* axes by 10%.

**Fig 29:**
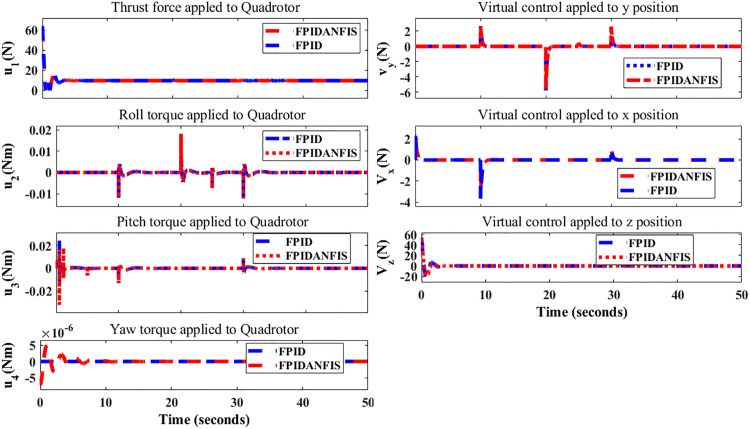
Control effort for triangular trajectory tracking in the presence of input disturbances.

**Fig 30:**
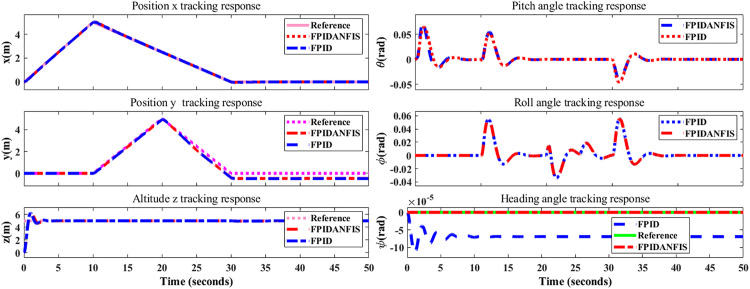
Position tracking response for triangular trajectory tracking in the presence of input disturbances.

**Fig 31:**
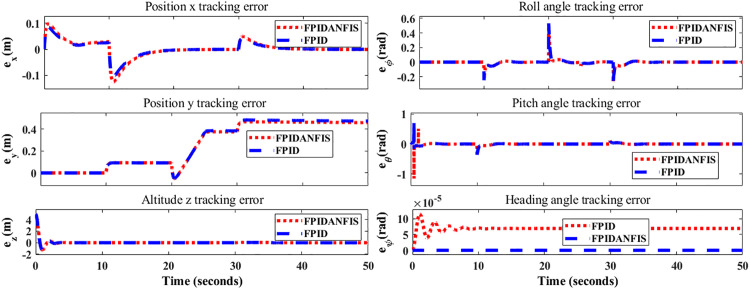
Tracking error for triangular trajectory tracking in the presence of input disturbances.

**Fig 32:**
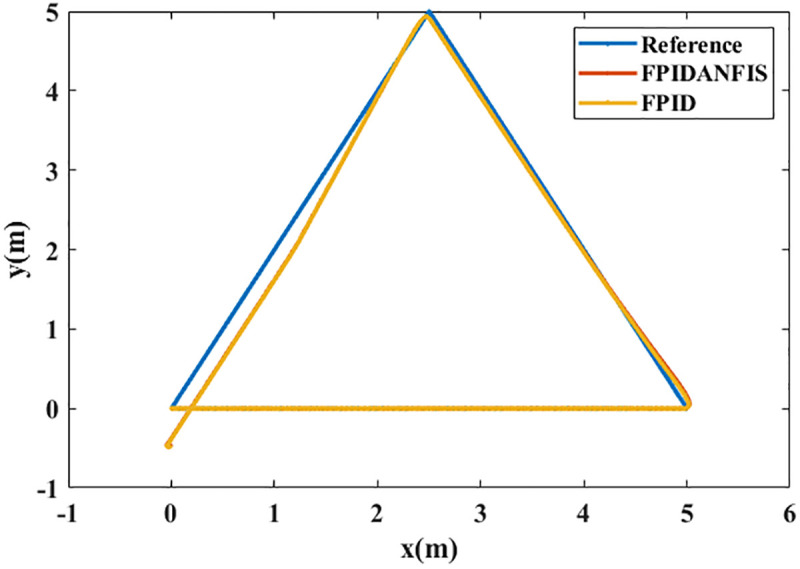
xy tracking response for triangular trajectory tracking in the presence of input disturbances.

**Fig 33:**
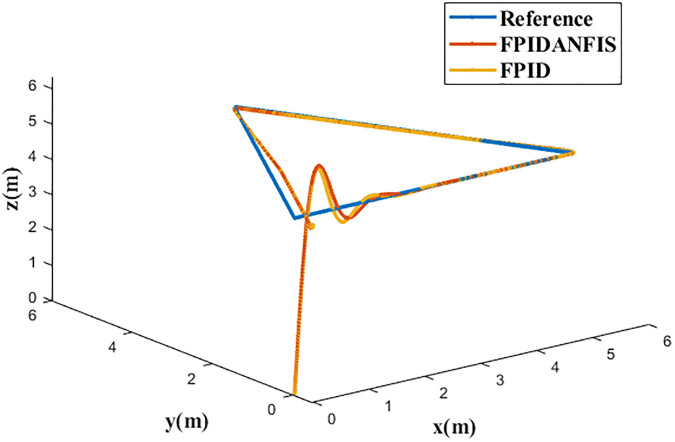
3D plot for triangular trajectory tracking in the presence of input disturbances.

The applied disturbance force was set to 0.5 N in the *x*, *y*, and *z* directions during specific time intervals: 5–10 seconds for the *x* axis, 20–25 seconds for the *y* axis, and 30–35 seconds for the *z* axis. These conditions were designed to rigorously evaluate the system’s performance under external disturbances and dynamic parameter changes for triangular trajectory. The simulation results for triangular trajectory, incorporating the effects of the input disturbances are presented in Figs 29, 30, 31, 32, and 33.

**Fig 34:**
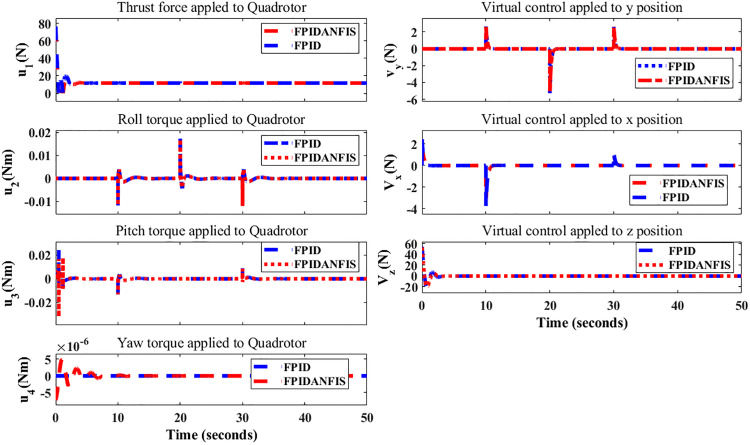
Control effort for triangular trajectory tracking in the presence of parameter variation.

**Fig 35:**
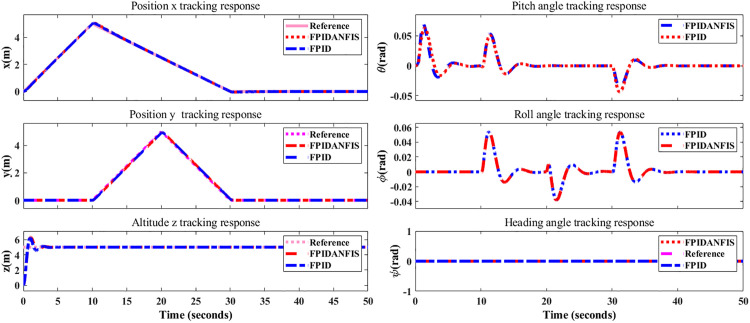
Position tracking response for triangular trajectory tracking in the presence of parameter variation.

**Fig 36:**
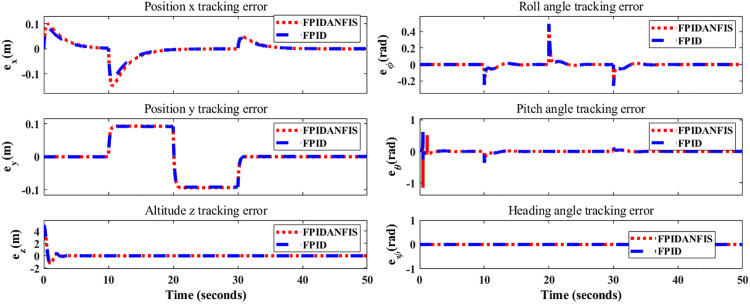
Tracking error for triangular trajectory tracking in the presence of parameter variation.

**Fig 37:**
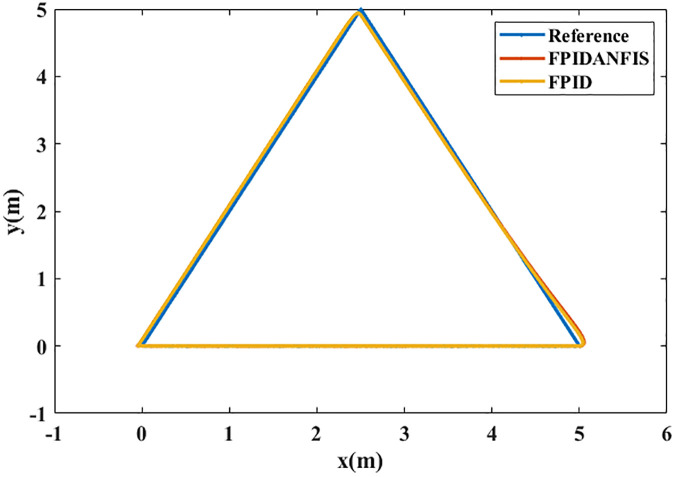
xy tracking response for triangular trajectory tracking in the presence of parameter variation.

**Fig 38:**
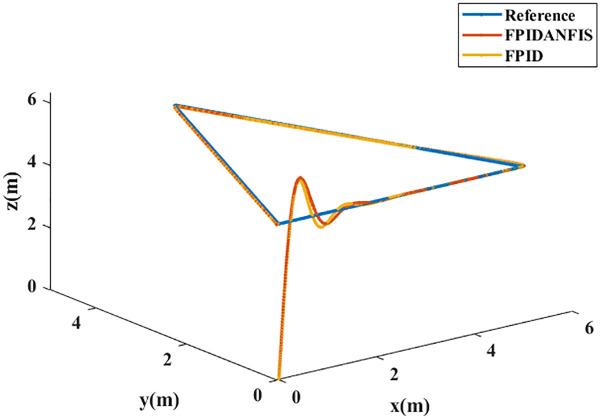
3D plot for triangular trajectory tracking in the presence of parameter variation.

The simulation results for triangular trajectory, incorporating the effects of the variation in parameter are presented in Figs 34, 35, 36, 37 and 38.

## 7 Comparison of controllers for triangular trajectory based on performance criteria

This study undertakes a comprehensive comparison of two controllers— Fuzzy Proportional-Integral-Derivative (FPID) and proposed FPIDANFIS controller based on performance criteria in the tabular form described in Tables 8–10 in undisturbed conditions, disturbed(with input disturbance added) and variation in parameter conditions respectively.

**Table 8:** Comparison of controllers using integral time absolute error (ITAE) criteria for triangular trajectory(undisturbed condition).

States	FPID (ITAE)	FPIDANFIS (ITAE)
*x*	8.456	7.945
*y*	34.326	31.457
*z*	4.145	3.927
ϕ	9.875	8.459
θ	4.445	3.875
ψ	0	0

**Table 9:** Comparison of controllers using integral time absolute error (ITAE) criteria for triangular tracking in the presence of input disturbances.

States	FPID (ITAE)	FPIDANFIS (ITAE)
*x*	9.885	9.779
*y*	465.7	270.2
*z*	12.21	9.91
ϕ	11.2	11.16
θ	5.86	5.845
ψ	0	0

**Table 10:** Comparison of controllers using integral time absolute error (ITAE) criteria for triangular tracking in the presence of parameter variation.

States	FPID (ITAE)	FPIDANFIS (ITAE)
*x*	10.04	9.949
*y*	37.29	37.24
*z*	5.902	4.594
ϕ	10.34	10.32
θ	6	5.978
ψ	0	0

### 7.1 Analysis and discussion of controller performance

The performance of the FPID and FPIDANFIS controllers is evaluated under three scenarios: undisturbed conditions, input disturbances, and parameter variations. The analysis is based on the Integral Time Absolute Error (ITAE) criteria as shown in Tables 8–10.

#### 7.1.1 Undisturbed conditions.

From Table 8, it is observed that the FPIDANFIS controller consistently outperforms the FPID controller across all states in undisturbed conditions. The ITAE values are lower for FPIDANFIS in every state, indicating superior tracking performance. For example:

- In the *x* state, FPIDANFIS achieves an ITAE of 7.945 compared to 8.456 for FPID.
- The most significant improvement is observed in the *y* state, where FPIDANFIS achieves an ITAE of 31.457 compared to 34.326 for FPID.

The FPIDANFIS controller exhibits its strengths in handling complex nonlinearities and dynamic conditions more effectively than FPID. However, both controllers perform equally for the ψ state, which may suggest limited control effort is required for this state.

#### 7.1.2 Performance under input disturbances.

In the presence of input disturbances Table 9, FPIDANFIS again outperforms FPID, particularly in the *y* and *z* states:

- For the *y* state, FPIDANFIS achieves a dramatic improvement with an ITAE of 270.2 compared to 465.7 for FPID.
- In the *z* state, FPIDANFIS records an ITAE of 9.91, whereas FPID has an ITAE of 12.21.

These results demonstrate the robustness of FPIDANFIS under external disturbances. However, the performance differences in other states such as ϕ and θ are minimal, which indicates similar control effectiveness in these states under disturbances. For the ψ state, the ITAE remains zero for both controllers.

#### 7.1.3 Performance under parameter variations.

In scenarios with parameter variations Table 10, the FPIDANFIS controller continues to show superior performance.

- For the *z* state, FPIDANFIS achieves a significant reduction in ITAE (4.594 vs. 5.902 for FPID).
- The *x* and *y* states exhibit minor performance differences, with FPIDANFIS slightly outperforming FPID (e.g., 9.949 vs. 10.04 in *x*).

Despite these strengths, the improvements are marginal in some states such as ϕ and $\begin{array}{c} \theta \end{array}$, which suggests limitations in FPIDANFIS when parameter variations are less critical.

### 7.2 Limitations of controllers

Both controllers exhibit limitations in specific scenarios:

- The FPID controller struggles significantly in handling disturbances, particularly in the *y* state, where its ITAE values are considerably higher.
- While FPIDANFIS shows improved performance, its benefits are more pronounced in specific states, such as *z* and *y*, and less noticeable in others (ϕ, θ, and ψ).
- The yaw reference is constant in all test scenarios. Therefore, the zero ITAE values reflect the simplicity of the tracking task rather than superior controller performance.

### 7.3 Summary of analysis and discussion of controllers performance

Overall, the FPIDANFIS controller demonstrates better robustness and tracking performance compared to FPID in all tested scenarios. However, its advantages are more prominent under challenging conditions, such as disturbances and parameter variations. The minimal differences in some states highlight areas for potential optimization in the FPIDANFIS design (Table 11).

**Table 11:** Summary of strengths and limitations of FPIDANFIS controller compared with FPID.

Strengths	Limitations
Lower ITAE values in undisturbed conditions across all states, indicating improved trajectory tracking.	Minimal improvement in rotational states (ϕ, θ) compared to FPID.
Significant reduction in ITAE for *y* state under input disturbances (approximately 42% improvement).	Limited adaptation in *y* state under parameter variations, with only a marginal performance gain.
Better robustness to parameter variations, especially in the *z* state (22.12% ITAE reduction).	Requires additional tuning to enhance rotational state response.
Consistently superior performance in translational states (*x*, *y*, *z*), suggesting better adaptability.	Performance improvements in ϕ and θ are relatively small under disturbance and parameter variations.

### 7.4 Limitations of the study

While the proposed FPIDANFIS controller demonstrates improved performance, the study has the following limitations:

- **Computational Complexity:** The ANFIS-based controller requires extensive computations for real-time adaptation, which may pose challenges for resource-constrained systems.
- **Hardware Implementation Constraints:** The study is limited to simulations, and real-time deployment on UAV hardware may introduce additional challenges, such as processing delays and sensor noise.
- **Potential Scalability Issues:** The effectiveness of the controller has been validated for a single UAV system, and its scalability to multi-UAV or larger autonomous systems requires further investigation.

## 8 Conclusion and future work

This paper presents a novel control strategy for quadrotor UAV trajectory tracking, demonstrating enhanced accuracy and robustness across a range of operating conditions. The novelty of the proposed approach lies in leveraging historical data from a baseline FPID controller to design the new FPIDANFIS controller. Specifically, input–output data from the FPID, with the tracking error and its derivative as inputs and the FPID control signals as outputs, were used to train an ANFIS model, which then generates the adaptive FPIDANFIS controller. Comparative analysis shows that FPIDANFIS consistently outperforms the baseline FPID, effectively handling disturbances and system uncertainties. While the approach significantly improves adaptability and reliability, further refinements are needed to enhance control consistency and extend applicability to real-world UAV operations. Future work will focus on improving the controller’s adaptability and validating its performance under practical flight conditions.

## References

[1] Shakhatreh H, Sawalmeh AH, Al-Fuqaha A, Dou Z, Almaita E, Khalil I, et al. Unmanned aerial vehicles (UAVs): a survey on civil applications and key research challenges. IEEE Access. 2019;7:48572–634. doi: 10.1109/access.2019.2909530

[2] Wandelt S, Wang S, Zheng C, Sun X. AERIAL: A meta review and discussion of challenges toward unmanned aerial vehicle operations in logistics, mobility, and monitoring. IEEE Trans Intell Transport Syst. 2024;25(7):6276–89. doi: 10.1109/tits.2023.3343713

[3] Le N-B-v, Thai H-D, Yoon C-W, Huh J-H. Recent development of drone technology software engineering: a systematic survey. IEEE Access. 2024;12:128729–5. doi: 10.1109/ACCESS.2024.3454546

[4] Ralegankar VK, Bagul J, Thakkar B, Gupta R, Tanwar S, Sharma G, et al. Quantum cryptography-as-a-service for secure uav communication: applications, challenges, and case study. IEEE Access. 2022;10:1475–92. doi: 10.1109/access.2021.3138753

[5] Dong S. Application research of quadrotor UAV motion planning system based on heuristic search and minimum-snap trajectory optimization. 2021 IEEE 5th Advanced Information Technology, Electronic and Automation Control Conference (IAEAC), Chongqing, China, 2021, 2270–4. 10.1109/IAEAC50856.2021.9390941

[6] Rathnayake N, Dang TL, Hoshino Y. Performance comparison of the ANFIS based quad-copter controller algorithms. In: 2021 IEEE International Conference on Fuzzy Systems (FUZZ-IEEE), 2021. 1–8. 10.1109/fuzz45933.2021.9494344

[7] Zadeh LA. Outline of a new approach to the analysis of complex systems and decision processes. IEEE Trans Syst, Man, Cybern. 1973;SMC-3(1):28–44. doi: 10.1109/tsmc.1973.5408575

[8] Mateo Sanguino TJ, Lozano Domínguez JM. Design and stabilization of a Coandă effect-based UAV: Comparative study between fuzzy logic and PID control approaches. Robotics and Autonomous Systems. 2024;175:104662. doi: 10.1016/j.robot.2024.104662

[9] Ali ZA, Wang D, Aamir M. Fuzzy-based hybrid control algorithm for the stabilization of a tri-rotor UAV. Sensors (Basel). 2016;16(5):652. doi: 10.3390/s16050652 27171084 PMC4883343

[10] Dorzhigulov A, Bissengaliuly B, Spencer BF Jr, Kim J, James AP. ANFIS based quadrotor drone altitude control implementation on Raspberry Pi platform. Analog Integr Circ Sig Process. 2018;95(3):435–45. doi: 10.1007/s10470-018-1159-8

[11] Housny H, Chater EA, Fadil HE. PSO-based ANFIS for quadrotor system trajectory-tracking control. In: 2020 1st International Conference on Innovative Research in Applied Science, Engineering and Technology (IRASET), 2020. 1–6. 10.1109/iraset48871.2020.9092015

[12] Mahfouz M, Kader SA. Quadrotor unmanned aerial vehicle controller design and synthesis. In: 2015 Tenth International Conference on Computer Engineering & Systems (ICCES), 2015. 60–6. 10.1109/icces.2015.7393019

[13] Gupta RA, Kumar R, Surjuse RS. ANFIS based intelligent control of vector controlled induction motor drive. 2009 Second International Conference on Emerging Trends in Engineering & Technology, Nagpur, India, 2009. 674–80. 10.1109/ICETET.2009.35

[14] Shaikh UA, AlGhamdi MK, AlZaher HA. Novel product ANFIS-PID hybrid controller for buck converters. 2018. doi: 10.1049/joe.2018.0113

[15] Al-Fetyani M, Hayajneh M, Alsharkawi A. Design of an executable ANFIS-based control system to improve the attitude and altitude performances of a quadcopter drone. Int J Autom Comput. 2021;18:124–40. doi: 10.1007/s11633-020-1251-2

[16] Jang J-SR. ANFIS: adaptive-network-based fuzzy inference system. IEEE Trans Syst Man Cybern. 1993;23(3):665–85. doi: 10.1109/21.256541

[17] Selma B, Chouraqui S, Abouaïssa H. Optimization of ANFIS controllers using improved ant colony to control an UAV trajectory tracking task. SN Appl Sci. 2020;2(5). doi: 10.1007/s42452-020-2236-z

[18] Chen M, Linkens DA. A hybrid neuro-fuzzy PID controller. Fuzzy Sets and Systems. 1998;99(1):27–36. doi: 10.1016/s0165-0114(96)00401-0

[19] Mukhopadhyay J, Choudhury S, Sengupta S. ANFIS based speed and current controller for switched reluctance motor. In: 2021 IEEE 4th International Conference on Computing, Power and Communication Technologies (GUCON), 2021. 1–6. 10.1109/gucon50781.2021.9573617

[20] Hasan MA, Mishra RK, Singh S. Speed control of DC motor using adaptive neuro fuzzy inference system based PID controller. 2019 2nd International Conference on Power Energy, Environment and Intelligent Control (PEEIC), Greater Noida, India, 2019. 138–43. 10.1109/PEEIC47157.2019.8976579

[21] Htun HN, Yakunin AN, Paing HS, Win K. Implementation of ANFIS controller for DC motor on an arduino due board. 2021 IEEE Conference of Russian Young Researchers in Electrical and Electronic Engineering (ElConRus), St. Petersburg, Moscow, Russia, 2021. 2668–71. 10.1109/ElConRus51938.2021.9396740

[22] Basha MG, Chenchireddy K, Sydu SA, Sultana W, Kumar V, Ganesh LB. Performance improvement of DC motor by using ANFIS controller. 2024 4th International Conference on Pervasive Computing and Social Networking (ICPCSN), Salem, India, 2024. 932–8. 10.1109/ICPCSN62568.2024.00157

[23] Kareem AA, Oleiwi BK, Mohamed MJ. Planning the optimal 3D quadcopter trajectory using a delivery system-based hybrid algorithm. International Journal of Intelligent Engineering and Systems. 2023;16(Issue.2):427–39. doi: 10.22266/ijies2023.0430.34

[24] Kareem AA, Oleiwi BK, Mohamed MJ. Optimal trajectory tracking: a comparative study of autonomous quadcopter controllers. Jordan Journal of Mechanical and Industrial Engineering. 2024;18(Issue 2):411–9. doi: 10.59038/jjmie/180213

[25] Jia K, Lin S, Du Y, Zou C, Lu M. Research on Route Tracking Controller of Quadrotor UAV Based on Fuzzy Logic and RBF Neural Network. IEEE Access. 2023;11:111433–47. doi: 10.1109/access.2023.3322944

[26] Santos M, de la Cruz JM, Dormido S, de Madrid AP. Between fuzzy-PID and PID-conventional controllers: a good choice. In: Proceedings of North American Fuzzy Information Processing. 123–7. 10.1109/nafips.1996.534716

[27] Satpathy S, Biswal AP. A comparative study between PID and fuzzy controller. 2021 1st Odisha International Conference on Electrical Power Engineering, Communication and Computing Technology(ODICON), Bhubaneswar, India, 2021. 1–4. 10.1109/ODICON50556.2021.9428994

[28] Can E. Energy-aware adaptive altitude control of UAVs via fuzzy–PSO optimization within a port-hamiltonian framework under icing and sensor noise. Int J Aeronaut Space Sci. 2025. doi: 10.1007/s42405-025-01087-2

[29] Can E. UAV DC Motor behaviors control using new hybrid PSO-fuzzy logic with a dynamic error based optimization. Aerospace Science and Technology. 2026;168:111156. doi: 10.1016/j.ast.2025.111156

[30] Liu K, Yang P, Wang R, Jiao L, Li T, Zhang J. Observer-Based Adaptive Fuzzy Finite-Time Attitude Control for Quadrotor UAVs. IEEE Trans Aerosp Electron Syst. 2023;59(6):8637–54. doi: 10.1109/taes.2023.3308552

[31] Ghasemi A, Azimi MM. Adaptive fuzzy PID control based on nonlinear disturbance observer for quadrotor. Journal of Vibration and Control. 2022;29(13–14):2965–77. doi: 10.1177/10775463221089734

[32] Yasmine Z, Karim B, Razika B, Younes B. Adaptive Fuzzy Logic Control of Quadrotor. IJRCS. 2024;4(4):2095–118. doi: 10.31763/ijrcs.v4i4.1583

[33] Madebo NW. Hybrid adaptive PID control strategy for UAVs using combined neural networks and fuzzy logic. PLoS One. 2025;20(8):e0331036. doi: 10.1371/journal.pone.0331036 40880437 PMC12396714

[34] Molapo M, Twala B. Neuro-fuzzy-based adaptive control for autonomous drone flight. Qeios. 2023. doi: 10.32388/V4GY5T

[35] Song W, Li Z, Xu B, Wang S, Meng X. Research on Improved Control Algorithm of Quadrotor UAV based on Fuzzy PID. 2022 IEEE International Conference on Artificial Intelligence and Computer Applications (ICAICA), Dalian, China, 2022. 361–5. 10.1109/ICAICA54878.2022.9844554

[36] Yu Y, Chen C, Guo J, Chadli M, Xiang Z. Adaptive Formation Control for Unmanned Aerial Vehicles With Collision Avoidance and Switching Communication Network. IEEE Trans Fuzzy Syst. 2024;32(3):1435–45. doi: 10.1109/tfuzz.2023.3327114

[37] Muthusamy PK, Garratt M, Pota H, Muthusamy R. Real-Time Adaptive Intelligent Control System for Quadcopter Unmanned Aerial Vehicles With Payload Uncertainties. IEEE Trans Ind Electron. 2022;69(2):1641–53. doi: 10.1109/tie.2021.3055170

[38] Lin J, Liu X, Ren Z. Fuzzy PID Control for Multi-joint Robotic Arm. In: 2022 IEEE 20th International Conference on Industrial Informatics (INDIN), 2022. 723–8. 10.1109/indin51773.2022.9976122

[39] Ibrahim MK, Sajid A, Ullah I, Ali T, Ayaz M, Aggoune E-HM. Artificial Neural Fuzzy Inference Rule-Based (ANFIS) Model for Offloading Tasks for Edge, Cloud, and UAVs Environment. IEEE Access. 2024;12:154443–54. doi: 10.1109/access.2024.3483656

[40] Kurnaz S, Kaynak O, Konakoğlu E. Adaptive neuro-fuzzy inference system based autonomous flight control of unmanned air vehicles. Lecture Notes in Computer Science. Springer Berlin Heidelberg. 2007. 14–21. doi: 10.1007/978-3-540-72383-7_3

[41] Esper IM, Rosa PFF. Heading controller for a fixed wing UAV with reduced control surfaces based on ANFIS. In: 2015 IEEE International Conference on Dependable Systems and Networks Workshops, 2015. 118–23. 10.1109/dsn-w.2015.27

[42] Elbatal A, Elkhatib MM, Youssef AM. Intelligent autopilot design based on adaptive neuro -fuzzy technique and genetic algorithm. 2020 12th International Conference on Electrical Engineering (ICEENG), Cairo, Egypt, 2020. 377–82. 10.1109/ICEENG45378.2020.9171702

[43] Mahfouz M, Hafez AT, Ashry M, Elnashar GA. Backstepping-ANFIS hybrid controller design for cooperative quadrotors. J Appl Res Technol. 2023;21(4):665–87. doi: 10.22201/icat.24486736e.2023.21.4.1738

[44] Vargas OS, De León Aldaco SE, Alquicira JA, Vela-Valdés LG, Núñez ARL. Adaptive network-based fuzzy inference system (ANFIS) applied to inverters: a survey. IEEE Trans Power Electron. 2024;39(1):869–84. doi: 10.1109/tpel.2023.3327014

[45] Sharma P, Ohri J. ANFIS based PID control of antilock braking system model. 2023 7th International Conference on Computer Applications in Electrical Engineering-Recent Advances (CERA), Roorkee, India, 2023. 1–6. 10.1109/CERA59325.2023.10455135

[46] Islam MdA, Singh JG, Jahan I, Lipu MSH, Jamal T, Elavarasan RM, et al. Modeling and performance evaluation of ANFIS controller-based bidirectional power management scheme in plug-in electric vehicles integrated with electric grid. IEEE Access. 2021;9:166762–80. doi: 10.1109/access.2021.3135190

[47] Gomez WRG, Caballero JM. Development of ANFIS-based controller for spray drying machine. In: 2023 8th International Conference on Business and Industrial Research (ICBIR), 2023. 244–9. 10.1109/icbir57571.2023.10147495

[48] Lu Q, Ren B, Parameswaran S. Uncertainty and disturbance estimator-based global trajectory tracking control for a quadrotor. IEEE/ASME Trans Mechatron. 2020;25(3):1519–30. doi: 10.1109/tmech.2020.2978529

[49] Antonelli G, Cataldi E, Arrichiello F, Robuffo Giordano P, Chiaverini S, Franchi A. Adaptive trajectory tracking for quadrotor MAVs in presence of parameter uncertainties and external disturbances. IEEE Trans Contr Syst Technol. 2018;26(1):248–54. doi: 10.1109/tcst.2017.2650679

[50] Lopez-Sanchez I, Rossomando F, Pérez-Alcocer R, Soria C, Carelli R, Moreno-Valenzuela J. Adaptive trajectory tracking control for quadrotors with disturbances by using generalized regression neural networks. Neurocomputing. 2021;460:243–55. doi: 10.1016/j.neucom.2021.06.079

[51] Wu X, Liu Y. Trajectory tracking control of quadrotor UAV. 2018 37th Chinese Control Conference (CCC), Wuhan, China, 2018. 10020–5. 10.23919/ChiCC.2018.8482939

[52] Tian Y-X, Li C, Yu W-T, Xiong J-J. Adaptive position and attitude trajectory tracking control for a quadrotor UAV. 2022 International Conference on Automation, Robotics and Computer Engineering (ICARCE), Wuhan, China, 2022. 1–4. 10.1109/ICARCE55724.2022.10046429

[53] Gu Y, Yuan C. Adaptive robust trajectory tracking control of fully actuated bipedal robotic walking. In: 2020 IEEE/ASME International Conference on Advanced Intelligent Mechatronics (AIM), 2020. 1310–5. 10.1109/aim43001.2020.9158814

[54] Gao M, Li J, Hu T, Luo J, Feng B. Intelligent vehicle trajectory tracking with an adaptive robust nonsingular fast terminal sliding mode control in complex scenarios. Sci Rep. 2024;14(1):31085. doi: 10.1038/s41598-024-82021-6 39730610 PMC11681002

[55] Indayu N, Darwito PA. ANFIS-SMC for trajectory tracking and obstacle avoidance control of quadcopter. In: 2023 8th International Conference on Instrumentation, Control, and Automation (ICA), 2023. 230–5. 10.1109/ica58538.2023.10273124

[56] Hou Y, Chen D, Yang S. Adaptive robust trajectory tracking controller for a quadrotor UAV with uncertain environment parameters based on backstepping sliding mode method. IEEE Trans Automat Sci Eng. 2025;22:4446–56. doi: 10.1109/tase.2023.3324434

[57] Maurya H, Behera L, Verma N. Trajectory tracking of quad-rotor UAV using fractional order $PI^{\mu }D^{\lambda }$ controller. doi: 10.1007/978-981-13-1132-114

[58] Madebo NW, Abdissa CM, Lemma LN. Enhanced trajectory control of quadrotor UAV using fuzzy PID based recurrent neural network controller. IEEE Access. doi: 10.1109/ACCESS.2024.3516494

[59] Lee T, Leok M, McClamroch NH. Nonlinear robust tracking control of a quadrotor UAV on SE(3). 2012 American Control Conference (ACC), Montreal, QC, Canada, 2012. 4649–54. 10.1109/ACC.2012.6315143

[60] Kazemi MH, Tarighi R. PID-based attitude control of quadrotor using robust pole assignment and LPV modeling. Int J Dynam Control. 2024;12(7):2385–97. doi: 10.1007/s40435-023-01372-6

[61] Bresciani T. Modelling, identification and control of a quadrotor helicopter. 2008.

[62] Cheng W, Hou Z. Attitude control of quadrotor UAV based on adaptive sliding mode control. 2022 34th Chinese Control and Decision Conference (CCDC), Hefei, China, 2022. 5878–84. 10.1109/CCDC55256.2022.10033888

[63] Johan P, Gravdahl JT, Pettersen JY. Vehicle-manipulator systems: modeling for simulation, analysis, and control advances in industrial control. Springer Science & Business Media. 2013.

[64] Al-Hmouz A, Jun S, Al-Hmouz R, Jun Y. Modeling and simulation of an adaptive neuro-fuzzy inference system (ANFIS) for mobile learning. IEEE Trans Learning Technol. 2012;5(3):226–37. doi: 10.1109/tlt.2011.36

[65] Jang JSR, Sun CT, Mizutani E. Neuro-Fuzzy and Soft Computing. Prentice Hall. 1997.

[66] Madebo NW. Enhancing intelligent control strategies for UAVs: A comparative analysis of fuzzy logic, fuzzy PID, and GA-optimized fuzzy PID controllers. IEEE Access. 2025;13:16548–63. doi: 10.1109/ACCESS.2025.3532743

[67] Sureshkumar V, Cohen K. Autonomous Control of a Quadrotor UAV using Fuzzy Logic. University of Cincinnati: Cincinnati, Ohio, United States. https://www.researchgate.net/publication/271550748

[68] Abbasi E, Mahjoob M, Yazdanpanah R. Controlling of quadrotor uav using a fuzzy system for tuning the pid gains in hovering mode. In 10th Int. Conf Adv Comput Entertain Technol. 1–6, 2013.

[69] Wang L. Building structure control system application research based on fuzzy logic algorithm. IEEE Access. 2023;11:144799–811. doi: 10.1109/ACCESS.2023.3342632

[70] Mastacan L, Olah I, Anita LI, Peiu T. Fuzzy logic controllers design: a case study. In: Proceedings of IEEE International Conference on Intelligent Engineering Systems. 233–8. 10.1109/ines.1997.632422

[71] Huan T, Huang D, Luo D. Attitude Tracking for a Quadrotor UAV Based on Fuzzy PID Controller. 2018. doi: 10.1109/ICCSS.2018.8572353

[72] Zareb M, Ayad R, Nouibat W. Fuzzy-PID hybrid control system to navigate an autonomous mini-Quadrotor. In: 3rd International Conference on Systems and Control, 2013. 906–13. 10.1109/icosc.2013.6750965

[73] Xue G, Chang Q, Wang J, Zhang K, Pal NR. An adaptive neuro-fuzzy system with integrated feature selection and rule extraction for high-dimensional classification problems. IEEE Trans Fuzzy Syst. 2023;31(7):2167–81. doi: 10.1109/tfuzz.2022.3220950

[74] Yuningsih L, Huizen RR, Pradipta GA, Ayu PDW, Hostiadi DP. Adaptive Neuro-Fuzzy Inference System For Medical Image Classification -A Review. 2022 4th International Conference on Cybernetics and Intelligent System (ICORIS), Prapat, Indonesia, 2022. 1–9. 10.1109/ICORIS56080.2022.10031548

[75] Devi S, Kumar S, Kushwaha GS. An adaptive neuro fuzzy inference system for prediction of anxiety of students. 2016 Eighth International Conference on Advanced Computational Intelligence (ICACI), Chiang Mai, Thailand, 2016. 7–13. 10.1109/ICACI.2016.7449795

[76] Sharkawy A-N, Bekhiti B. Enhanced estimation and short-term forecasting of output power of a solar photovoltaic plant using adaptive neuro-fuzzy inference system (ANFIS). Journal of Electrical Systems and Inf Technol. 2026;13(1). doi: 10.1186/s43067-025-00302-0

[77] Chen X, Zhang X. Nonlinear feedback control based on ANFIS. 2015 12th International Conference on Fuzzy Systems and Knowledge Discovery (FSKD), Zhangjiajie, China, 2015. 559–63. 10.1109/FSKD.2015.7382003

